# Concepts of health in different contexts: a scoping review

**DOI:** 10.1186/s12913-022-07702-2

**Published:** 2022-03-24

**Authors:** V. P. van Druten, E. A. Bartels, D. van de Mheen, E. de Vries, A. P. M. Kerckhoffs, L. M. W. Nahar-van Venrooij

**Affiliations:** 1 Jeroen Bosch Academy Research, Jeroen Bosch Hospital Jeroen Bosch Ziekenhuis, PO Box 90153, Henri Dunantstraat 1, ‘s Hertogenbosch, 5223 GZ the Netherlands; 2 Tranzo Scientific Center for Care and Wellbeing, Tilburg University, PO Box 90153, Professor Cobbenhagenlaan 125, Tilburg, 5000 LE the Netherlands; 3 TiSEM Department of Management, Tilburg University, Tilburg, the Netherlands; 4 Department of Nephrology, Jeroen Bosch Hospital, ‘s Hertogenbosch, the Netherlands; 5 Department of Geriatric Medicine, Jeroen Bosch Hospital, ‘s Hertogenbosch, the Netherlands

**Keywords:** Health, Health concept, Health definition, Positive health, Health-related concepts, Health perception, Perceived health, Scoping review

## Abstract

The rationale of our study was that the World Health Organization’s (WHO) definition of health from 1947 which includes “… *complete physical, mental and social wellbeing…”* does not fit the current societal viewpoints anymore. The WHO’s definition of health implies that many people with chronic illnesses or disabilities would be considered unhealthy and complete wellbeing would be utopian and unfeasible for them. This is no longer uniformly accepted. Many alternative concepts of health have been discussed in the last decades such as ‘positive health’, which focusses on someone’s capability rather than incapability,. However, the question remains whether a general health concept can guide all healthcare practices. More likely, health concepts need to be specified for professions or settings. The objective of our study was to create a structured overview of published concepts of health from different perspectives by conducting a scoping review using the PRISMA-ScR guideline. A literature search was conducted in Pubmed and Cinahl. Articles eligible for inclusion focussed on the discussion or the conceptualisation of health or health-related concepts in different contexts (such as the perspective of care workers’ or patients’) published since 2009 (the Dutch Health Council raised the discussion about moving towards a more dynamic perspective on health in that year). Seventy-five articles could be included for thematic analyses. The results showed that most articles described a concept of health consisting of multiple subthemes; no consensus was found on one overall concept of health. This implies that healthcare consumers act based on different health concepts when seeking care than care workers when providing care. Having different understandings of the concepts of health can lead to misunderstandings in practice. In conclusion, from every perspective, and even for every individual, health may mean something different. This finding stresses the importance that care workers’ and healthcare consumers’ meaning of ‘health’ has to be clear to all actors involved. Our review supports a more uniform tuning of healthcare between healthcare providers (the organisations), care workers (the professionals) and healthcare consumers (the patients), by creating more awareness of the differences among these actors, which can be a guide in their communication.

**Supplementary Information:**

The online version contains supplementary material available at 10.1186/s12913-022-07702-2.

## Introduction

The World Health Organisation’s (WHO) definition of health does not fit the current societal viewpoints anymore [1]. The WHO definition of health is formulated as *“Health is a state of complete physical, mental and social wellbeing and not merely the absence of disease or infirmity”* [2]*.* Due to the word ‘complete’ in this definition, many people would not be considered healthy, because of their chronic illnesses or disabilities [1, 3]. For them, complete wellbeing would be utopian and unfeasible [4]. This is no longer uniformly accepted. Perspectives on people with physical disabilities are changing; they are no longer seen as ‘unhealthy’. On the other hand, the focus has shifted to the fact that people, when they get a chronic illness or disability, do need to adapt to their new situation; being able to do this is part of the recently developed paradigm of ‘positive health’ [5].

Many alternative concepts of health have been discussed in the last decades in philosophical and policy-oriented health and medicine debates, changing from health as being free from disease to health as someone’s capabilities. Prominent concepts of health which have been widely discussed and criticized by philosophers were developed by Boorse, Nordenfelt, and Nussbaum, respectively. Boorse’s biostatistical theory of health is a purely descriptive quality of an organism [6], which focusses on the functioning of body parts and on physiological systems being free from disease [7]. Nordenfelt discharged Boorse’s biostatistical theory and focussed on the ‘second-order ability to achieve vital goals’ in which actions are oriented to achieve minimal happiness, being a condition that the person prefers [8]. Like Nordenfelt, also Nussbaum’s capability approach is about achieving a set of capabilities in things that are important in a person’s life [9]. However, Nordenfelt focusses on a person’s health relating to human flourishing and achieving vital goals, while Nussbaum focusses on defining components of a person’s life that equally reflect human dignity as well as being able to be and to do certain things [10]. More recently, the International Classification of Functioning, Disability and Health (ICF) focussed on performance as well as capacities taking a broader set of aspects into account: body functions, activity and participation, environmental and personal factors, and body structures [11, 12].

These broader views on health were further extended since the positive health concept was postulated by Huber et al. in 2011 [5, 12]. Positive health focusses on someone’s capability rather than incapability, which means that people with chronic diseases or disabilities are no longer automatically seen as ‘not healthy’. Besides, there is a clear focus on resilience and self-management in social, physical and emotional challenges [5, 12]*.* To further operationalise the concept of positive health, Huber et al. conducted survey research among several stakeholders, asking what they considered important aspects of health. This resulted in the identification of 32 aspects categorized into six dimensions: 1) bodily functions, 2) mental functions and perception, 3) spiritual/existential dimension, 4) quality of life, 5) social and societal participation, and 6) daily functioning [12]. This concept has had a strong influence on healthcare policy in the Netherlands. Furthermore, since 2020 the Eastern Institute of Health (HSA) in Iceland has also started the implementation of positive health [13].

Reactions to the concept of positive health in the literature are mixed. The dimensions are seen as meaningful, however, the terms ‘adapt’ and ‘self-manage’ are being questioned. Jambroes et al. [14] discussed that several groups of people like frail elderly or people with mental disorders may not have the capacity to adapt or to manage their own health. Furthermore, giving people the responsibility for their own health management can cause people to feel guilty when health problems occur [14]. Prinsen and Terwee [15] tried to develop an instrument for measuring positive health. The results showed that the aspects of the ‘positive health’ concept had not yet been worked out clearly. The experts involved questioned whether the operationalisation of the conceptual model is a reflection of health or a reflection of aspects of life that influence health (i.e., are determinants of health) [15]. Also, Hafen [16] sees the ‘ability to adapt and self-manage’ as a determinant instead of part of the concept of ‘health’ itself. Motives for including aspects in the six dimensions were unclear, nor was it always clear to which dimension certain aspects belonged. Overlap was seen across aspects within dimensions [16].

It can be concluded that a clear alternative concept of health to replace the WHO definition has not yet been found. To our knowledge, no reviews have been conducted on this topic yet. However, it is important to have a clear and understandable general health concept for management, designing and redesigning policy, research and healthcare practices [5, 17]. It may help policymakers to establish and implement effective health policies to improve health status, quality of life, morbidity and mortality [18]. Clear understanding of the meaning of health by healthcare professionals and patients will foster active participation and will increase patient empowerment [18]. However, it is questionable whether a general health concept can guide all practices. More likely, health concepts need to be specified for specific professions or settings [1]. To answer this question, we conducted a scoping review, to create a structured overview of published concepts of health from different perspectives that can support a more uniform tuning of healthcare between healthcare providers and healthcare consumers. The research question was: How is the concept of health defined in different contexts and from different perspectives? (For example, from the perspective of healthcare providers and healthcare consumers).

## Method

### Design

This scoping review was conducted using the PRISMA-ScR guideline, which follows a systematic approach to map evidence and identify main concepts and theories on a topic [19]. This design was used because our research question was broad. In line with the design of a scoping review, our review did not have the intention to perform a structured evaluation of the research quality, but focussed on all publications available about our topic.

### Eligibility criteria

Articles eligible for inclusion focussed on the discussion or conceptualisation of health or health-related concepts. We included original research articles (interview or focus group discussions in qualitative design studies, surveys and concept mappings, quantitative or mixed methods studies exploring the concept), but also literature reviews, books, and letters to the editor. We excluded intervention studies using health or wellbeing related terms as one of their outcome measures. These studies do not focus primarily on discussing the concept of health. Validation studies of questionnaires or instruments evaluating health or wellbeing related terms not primarily focussing on the concept or definition of health were also excluded. Articles needed to be published in English between 2009 (the Dutch Health Council raised the discussion about moving towards a more dynamic perspective on health [5, 12] in that year) and May 2020.

### Information sources

The search was conducted in two databases: Pubmed and Cinahl, on May 25, 2020. The search was conducted by the first author (VvD) and was peer reviewed within the research team. These databases were chosen because of their focus on social behaviour and medical sciences. A snowball method was conducted on the references of the collected articles. Finally, four experts in the field were asked for additional papers that might have been missed.

### Search

The exact search string for PubMed is shown in Table 1 and for Cinahl in Table 2.

**Table 1:** The search string as conducted in PubMed

	Search term	Variations of the search terms entered in pubmed	Field
OR	Health-related wellbeing	health-related wellbeing OR health-related well-being	[Title/abstract]
	Health perception	OR health perception OR health perceptions	[Title/abstract]
	Attitude to health	OR attitude to health OR attitude health	[Title/abstract]
	Health concepts	OR health concepts OR health concept	[Title/abstract]
	Conceptualisation of health	OR conceptualisation of health OR conceptualisation health OR conceptualization of health OR conceptualization health OR conceptualisations of health OR conceptualisations health OR conceptualizations of health OR conceptualizations health	[Title/abstract]
	Positive health	OR positive health	[Title/abstract]
	Dimensions of wellbeing	OR dimensions of well-being OR dimensions of wellbeing OR dimensions well-being OR dimensions wellbeing OR dimension of well-being OR dimension of wellbeing OR dimension well-being OR dimension wellbeing	[Title/abstract]
	Perceived health	OR perceived health	[Title/abstract]
AND	Concept	concept*	[Title/abstract]
	Definition	OR defin*	[Title/abstract]
NOT	Child	child*	[Title/abstract]
	Kid	OR kid*	[Title/abstract]
	Adolescent	OR adolescent*	[Title/abstract]
	Newborn	OR newborn*	[Title/abstract]
	Infant	OR infant*	[Title/abstract]
	Baby	OR baby OR babies	[Title/abstract]
	Animals	OR animals	[Title/abstract]
Filter		English	
		11 years

**Table 2:** The search string as conducted in Cinahl

	Search term	Variations of the search terms entered in pubmed	Field
OR	Health-related wellbeing	health-related wellbeing OR health-related well-being	[Title/abstract]
	Health perception	OR health perception OR health perceptions	[Title/abstract]
	Attitude to health	OR attitude to health OR attitude health	[Title/abstract]
	Health concepts	OR health concepts OR health concept	[Title/abstract]
	Conceptualisation of health	OR conceptualisation of health OR conceptualisation health OR conceptualization of health OR conceptualization health OR conceptualisations of health OR conceptualisations health OR conceptualizations of health OR conceptualizations health	[Title/abstract]
	Positive health	OR positive health	[Title/abstract]
	Dimensions of wellbeing	OR dimensions of well-being OR dimensions of wellbeing OR dimensions well-being OR dimensions wellbeing OR dimension of well-being OR dimension of wellbeing OR dimension well-being OR dimension wellbeing	[Title/abstract]
	Perceived health	OR perceived health	[Title/abstract]
AND	Concept	concept*	[Title/abstract]
	Definition	OR defin*	[Title/abstract]
Filter		English	
		11 years	

### Selection of sources of evidence

Results of the search were uploaded in Rayyan, a free web application for independent selection of articles by multiple researchers. Two researchers (VvD and EB) independently screened all titles, abstracts and full-text articles for in- or exclusion. In addition, they discussed the articles on which there was disagreement. If no agreement was reached after discussion, a third researcher (LN-vV) was asked. Simultaneously, three senior researchers (LN-vV, EdV, DvdM) independently screened 10 % of the articles for in- or exclusion in the first two phases, the title and abstract selection, in order to validate the process.

### Data items

Preceding the coding process, a list of themes of interest was developed in consensus by the research team based on the aim of the scoping review and research question consisting of: 1*) concept of health* (a description of a health (−related) concept or definition, or what a health (−related) concept or definition should contain); 2) *dimensions of health* (category of health indicators for operationalisation in healthcare); 3) *perspective* (the perspective from which the concept of health was explored or the article written).

### Data charting process

For data extraction and synthesis, a thematic analysis was conducted to identify patterns within the data. First, a form including characteristics of the article and the list of themes was developed. The characteristics consisted of: country, article type/study design and perspective population/theoretical approach. The list of themes of interest was pilot tested on three articles by the first (VvD) and the last author (LN-vV). Second, the first author (VvD) started data extraction. Third, within the themes of interest, an open coding process was started using a bottom-up approach by the first author (VvD). The program ATLAS.ti (version 8) was used when coding the data. Codes were extracted from the data using the exact words from the original article. After coding all articles, the codes were categorised into potential subthemes, which fit into the overarching themes (i.e. *concept of health, dimensions of health, perspective*). We introduced a minimum level of appearance for subthemes in at least three articles as threshold for relevance. In case a subtheme was represented in at least 3 articles a description in detail of the subtheme was given. This threshold was based on consensus within the research team with the aim to keep our focus on the most relevant results. During the entire process, four researchers (EB, LN-vV, EdV, DvdM) were repeatedly consulted to discuss the analytic process and the development of the results.

### Synthesis of results

**Fig. 1:**
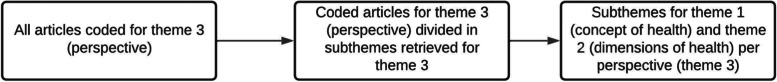
Diagram for synthesis of results

The articles were first divided into the retrieved subthemes for theme 3 (*perspective*), resulting in an overview of the results of theme 1 (*concept of health*) and theme 2 (*dimensions of health*) per subtheme of perspective (theme 3). In Fig. 1, the process for synthesis of results is shown.

## Results

### Selection of sources of evidence

**Fig. 2:**
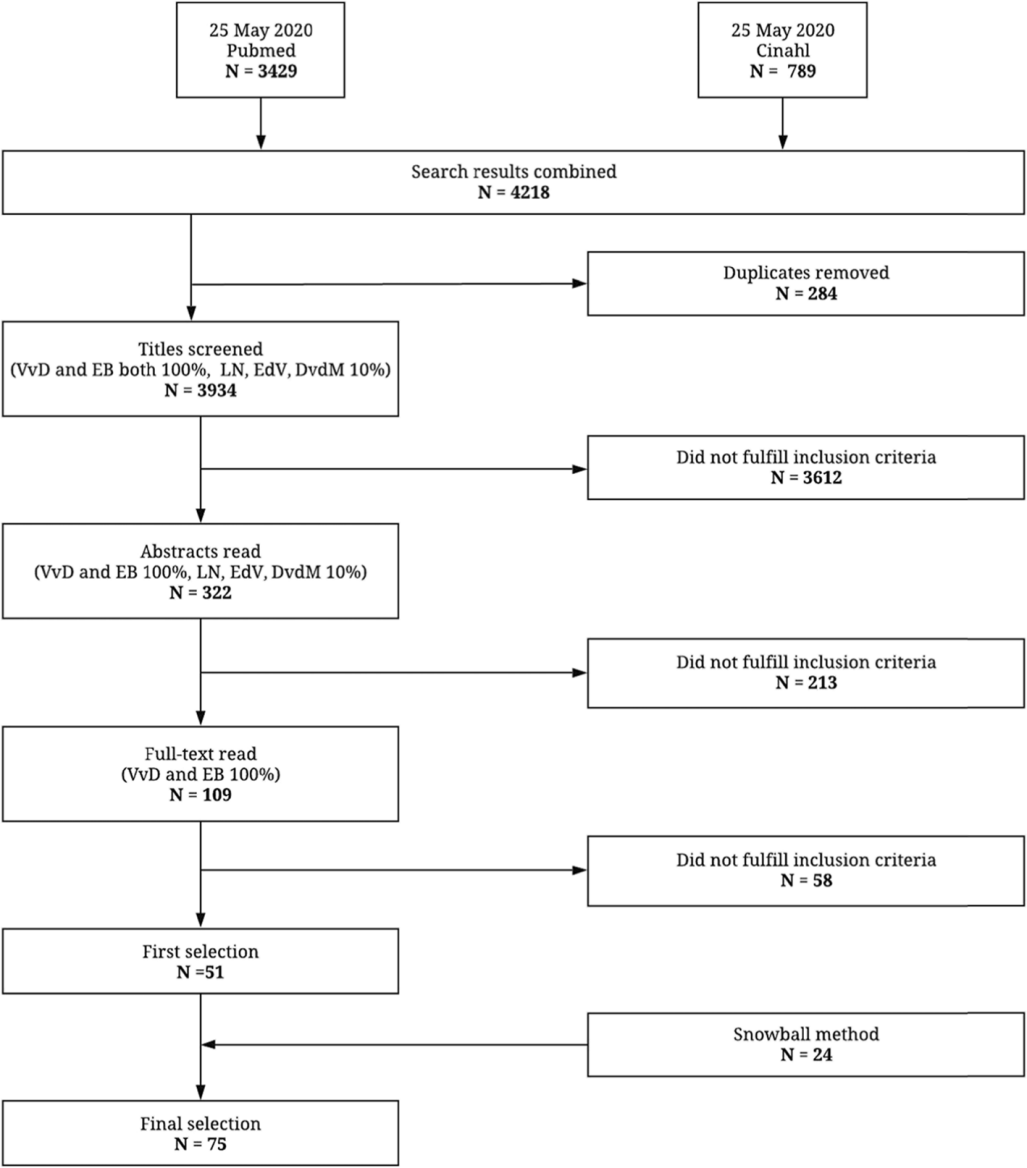
Flowchart for inclusion process

In Fig. 2, the flowchart with the number of retrieved articles in Pubmed and Cinahl and in−/exclusion per selection step is shown. Articles that did not fulfil the inclusion criteria after screening title, abstract or full text, respectively were not included for the next step. In the first step (title screening), there was an initial agreement of 94% between the authors VvD and EB. Simultaneously, the initial agreement with the senior researchers (LN-vV, EdV, DvdM) was 94%. In the second step (abstract screening), the initial agreement was 77% between the authors VvD and EB. In addition, the initial agreement with the senior researchers (LN-vV, EdV, DvdM) was 82%. In the third step (full-text screening), the initial agreement was 87% between the authors VvD and EB. In total, 75 articles were included for thematic analysis. Fifty-six articles were excluded in full-text screening, because they did not meet the inclusion criteria: 29 articles were not focussing on the concept or definition of health, 12 articles were intervention studies using health or wellbeing related terms as one of their outcome measures, 4 articles focussed on validation studies of questionnaires or instruments evaluating health or wellbeing related terms, for 8 articles no full texts were available, 2 articles were excluded because they were duplicates and 1 article was in Spanish.

### Characteristics of sources of evidence

For theme 1 (*concept of health)* 159 codes (210 quotes) were created during the analysis process. For theme 2 *(dimensions of health)* 72 codes (148 quotes) were created. For theme 3 *(perspective)* 68 codes (92 quotes) were created. Table 3 shows the coding scheme with the identified subthemes and codes of theme 1 *concept of health*. Table 4 shows the coding scheme with the identified subthemes and codes of theme 2 *dimensions of health*. To see details of Table 3 the supplementary Table 1 shows the same coding scheme, but includes also all related quotations from the 75 articles.

**Table 3:** The coding scheme; identified subthemes and codes for theme 1, the concept of health

Subtheme (explanation)	Codes			
Complete wellbeing or functioning (Functioning without any disturbance of diseases or infirmities)	Absence of disease and functioning	Absence of disease or illness	Absence of health problems	Adopting the biomedical view
	Biomedical interpretation of health	Complete physical	Getting off or maintaining desistance from harmful substance	Health as a condition to be fixed
	Health merely as absence of disease or infirmity	No tension	Normal functional ability	Normal physiological functional ability
	Not getting sick	Theoretical health is value free		
Wellbeing (Wellbeing in several ways but not referring to complete wellbeing or functioning)	Liberating and expansive way of being	Overall wellbeing	Physical-psychological wellbeing	Positive concept of wellbeing
	Sense of wellbeing	Spiritual and emotional wellbeing	State of wellbeing	Subjective wellbeing
	Wellbeing			
Adapting to change (Being able to adapt to personal or environmental health-related changes and circumstances)	Ability to adapt	Acceptance and adjustment with optimism	Adapt and accept limitations as part of ageing	Adaptation to worsening life conditions
	Adaptive system	Balance among dimensions	Dynamic nonlinear interaction	Dynamic over time
	Emotional balance	Flow of energy, listening to and respecting its rhythms	Functional adaptation	Health and peace are dynamic
	Health as a process	Health as a state of balance	Health can be fleeting both lost and regained	Health is a dynamic state
	Interactions	Maximal functional adaptation to illness or disability	Never-ending system of events	Overcoming health problems
	Process individuals go through during illness and health	Rhythmic pattern of living	Subject to change	
Multi-sided (Health is not related only to the physical dimension, but involves several dimensions)	Extends beyond the physical	Health as complex system	Health as comprehensive view	Health as holistic
	Health is not merely the absence of disease or infirmity	Health is not only normal physical function	Mind, body, soul or spirit concept	More than physical
	More than the absence of disease or illness	Multi-facetted concept	Multidimensional	Multidimensional, complex, elusive
	Not just focus on illness/disease elimination	Not merely the absence of problems	Person is more than his illness	Salutogenic health concept
	Tied to quality of life concept			
Self-management (Having self-control in life and in the health process)	Ability to do something independently	Ability to handle daily life activities	Ability to make health-related decisions	Ability to self-manage
	Absence or management of symptoms	Action and repetition of action in the health process	Autonomy	Autonomy and independency
	Being able to trust one’s ability	Capability to cope and manage malaise and wellbeing conditions	Control their lives	Experiencing enough energy in their own world
	Focus on a person’s strength	Independence	Manage daily activities	Manage one’s daily tasks
	Positive thinking and resourcefulness	Responsibility for yourself and others	Self-acceptance	Self-control
	Self-esteem	Self-esteem, self-concept	To be aware of one’s worth	To feel secure in oneself
Participation (Being active and participating in life)	Ability to be active and participating	Ability to live an active life	Being able to work	Being able to perform activities of daily living
	Capacity to perform tasks and fulfil societal roles	Dynamic participation in the world	Health as basic necessity or requirements to engage in activities	Participating in daily life
	Participation			
Satisfying life (Values that contribute satisfaction in life)	Ability to flourish	Ability to live a life that makes sense	Ability to satisfy by themselves the needs of daily live	Ability to take care of children
	Attitude towards life	Being in the world	Capacity to realize creaturely flourishing	Caring for others
	Connectedness with others	Contextual features of human society	Experience harmony in life	Experience meaningfulness in life
	Feel hope for the future	Good social contacts	Have a peaceful and positive feeling inside	Health as a commodity
	Health as a value	Health is about the whole life	Life satisfaction	Life worthy of equal human dignity
	Optimism	Peace in the family	Presence of multiple life satisfactions	Purpose in life
	Relationships with family	Social life satisfactory	Suffering as natural part of life	To live the good life
	Understanding of the goods, goals, and ends of human life			
Subjective (Personal perceptions and experiences about health)	Bodily phenomena	Current feelings	Disability is a state or experience of individuals	Enhancing personal strength
	Existential and subjective perspective of human experience	Experience of the being	Health as a resource for daily living	Health beliefs
	Health is based on individual and collective understandings of everyday realities	Health is subjective	Perceived health	Personal and social resources
	Personal evaluation of wellbeing	Personal experience	Person-centred and society-centred perspectives and values	Phenomenological ontology
	Self-perception	Subjective experience	Subjective features of human valuing	Subjective state
	Subjective wellbeing			
Daily functioning (Daily functioning in life)	Ability to achieve a basic cluster of beings and doings	Avoiding undesirable responses	Do what we always do	Functional health
	Functional states	Functionalist	Functionality and ability	Functioning
	Functioning in everyday life	Having desired emotional, cognitive, behavioural responses	Health-related behaviour	Mental health and functioning
	Objective features of human biology

**Table 4:** The coding scheme; identified subthemes and codes for theme 2, dimensions of health

Subtheme (explanation)	Codes			
Physical	Physical	Biomedical	Bodily functioning	Physical health
	Physical wellbeing	Somatic	Physical functioning	Physiological
Mental	Cognitive	Emotional	Emotional wellbeing	Mental function and perception
	Mental health	Mental wellbeing	Mental / emotional health	Mental phenomena
	Mental	Psychological	Psychological wellbeing	Psyche
Social	Community	Familial	Family	Psychosocial
	Social life	Social wellbeing	Social	Social and societal participation
	Social phenomena	Social functioning	Social health	Social factors
Spiritual	Spiritual	Spiritual health	Spiritual wellbeing	Spiritual / existential
	Spirituality			
Environmental (Dimensions in the environment of the patient’s life)	Context	Economical	Environment	Environmental
	Environmental wellbeing	Family and genealogy	Family factors	Farm life
	Financial	Impact of colonisation	Land	Political
	Space	Time		
Functional	Behavioural	Bodily function	Daily functioning	Functional
	Functional health	Physical functioning	Semiotic	Social functioning
Individual (Dimensions related to individual experiences)	Individual	Individual determinants	Individual wellbeing	Lived body
	Personal	Personal factors		
Others (Dimensions which cannot be categorised into the previous subthemes)	Anthropological	Balanced diet	Overall quality of life	Quality of life
	Symptoms	Medical	Māori healing techniques	

### Themes 1 and 2: concepts of health and dimensions of health

From the data for theme 1 *(concepts of health)* 159 codes were extracted and categorised. Nine subthemes arose by categorising the codes: multi-sided, adapting to change, complete wellbeing or functioning, participation, daily functioning, wellbeing, satisfying life, self-management, and subjective (see Table 3). Most articles (58/75) described a *concept of health* consisting of multiple subthemes. From the data for theme 2 *(dimensions of health)* 72 codes were extracted and categorised. Eight subthemes arose by categorising the codes for this theme: physical, mental, social, spiritual, individual, environmental, functional, and other dimensions (see Table 4). Almost half of the articles (36/75) described multiple dimensions of health. Similarities and differences in subthemes between theme 1 (*concepts of health)* and theme 2 (*dimensions of health)* were seen, represented by the related subthemes (see Tables 5, 6, 7, 8, 9, 10 and 11). An overview of the presented concepts and dimensions of health in more detail can be found in Supplementary Tables 2A to 2G (S2A-S2G). An overview table of the numbers of articles representing subthemes identified in the articles for theme 1 and theme 2, respectively, grouped per subtheme of perspective (theme 3), can be found in Supplementary Tables 3A and 3B.

**Table 5:** Included articles discussing health from a general population perspective

Authors, year	Country	Article type/ study design	Perspective (population)	Subthemes of Concept of health	Subthemes of Dimensions of health
Abuelaish et al., 2020 [20]	Canada	Literature debate	NA	Multi-sided, adapting to change	Social, environmental
Amzat & Razum, 2014 [21]	Nigeria	Book chapter	NA	Multi-sided	
Conner et al., 2019 [22]	USA	Survey research	African American, Asian American, European American, and Latin American men and women of lower and higher socioeconomic status (SES)	Complete wellbeing or functioning	Functional, physical, mental, social, spiritual, others
Downey & Chang 2013 [23]	USA	Empirical mixed-method study	American adults	Multi-sided	
Frenk & Gómez-Dantés, 2014 [24]	USA, Mexico	Commentary	NA	Multi-sided	
Kaldjian, 2017 [25]	USA	Forum discussion	NA	Daily functioning, subjective, satisfying life	
Karimi & Brazier, 2016 [26]	Switzerland	Current opinion	NA	Daily functioning, wellbeing	
Lipworth et al., 2011 [27]	Australia	Qualitative literature review	NA	Adapting to change	Physical, spiritual, mental, social
Makoul et al., 2009 [28]	USA	Survey research	American adults	Participation, self-management, complete wellbeing or functioning	Physical, mental, social, spiritual, functional, others
Pietersma et al., 2014 [29]	The Netherlands	Three-stage Delphi-procedure	Patients, family members of patients, clinicians, scientific experts, and general population	Self-management, satisfying life, participation	Mental, social, physical
Shilton et al., 2011 [30]	Australia, France	Letter to the editor	NA	Self-management	
Thumboo et al., 2018 [31]	Singapore, Finland	Qualitative research design	General public in Singapore	Subjective, participation, multis-sided	Physical, mental, social, spiritual, environmental
Williamson et al., 2009 [32]	Canada	Literature study	NA	Subjective

**Table 6:** Included articles discussing health from a care workers perspective

Authors, year	Country	Article type/ study design	Perspective (population)	Subthemes of Concept of health	Subthemes of Dimensions of health
Alslman et al., 2017 [33]	Jordan	Concept analysis	NA	Multi-sided	Physical, mental, social
Ashcroft & van Katwyk, 2016 [34]	Canada	Participatory action research	Social work educators, practitioners and students	Multi-sided, wellbeing	Mental, physical, social, spiritual, environmental
Bąk-Sosnowska et al., 2017 [35]	Poland	Survey research	General practitioners	Subjective	
Huber et al., 2016 [12]	The Netherlands	Mixed method study, qualitative approach, quantitative approach	Physicians, physiotherapists, policymakers, insurers, public health professionals, researchers, nurses, patients	Adapting to change, self-management, multi-sided	Functional, physical, mental, social, spiritual, others
Hunter et al., 2013 [36]	Australia	Phenomenography method	Patients and practitioners in integrative medicine clinic	Complete wellbeing or functioning, wellbeing, multi-sided	
Johansson et al., 2009 [37]	Sweden	Qualitative research design	Swedish health professionals	Multi-sided, subjective, satisfying life	Mental, physical, spiritual
Jormfeldt, 2009 [38]	Sweden	Cross-sectional study	Patients and staff in mental health services	Satisfying life, self-management	
Lyon, 2012 [39]	USA	Book chapter, conceptual overview	NA	Complete wellbeing or functioning, subjective	
Merry, 2012 [40]	Canada	Literature study	NA	Adapting to change, multi-sided, subjective	
Pace et al., 2011 [41]	Italy	Grounded theory approach	Care workers from Italy, South-America, and Eastern Europe	Wellbeing, complete wellbeing or functioning, adapting to change, satisfying life	Mental, physical, individual, environmental

**Table 7:** Included articles discussing health from a patient’s perspective

Authors, year	Country	Article type/ study design	Perspective (population)	Subthemes of Concept of health	Subthemes of Dimensions of health
Bickenbach, 2013 [42]	Switzerland	Literature study	Persons with disabilities	Subjective, daily functioning	
Ebrahimi et al., 2012 [43]	Sweden, USA	Phenomenological approach	Elders in emergency treatment, 80 years and older, or 65 years and older with chronic diseases	Subjective, adapting to change	Individual, environmental
Gorecki et al., 2010 [44]	United Kingdom	Review of the literature and qualitative approaches	patients with pressure ulcers		Physical, mental, functional, social, others
Huber et al., 2016 [12]	The Netherlands	Mixed method study, qualitative approach, quantitative approach	Physicians, physiotherapists, policymakers, insurers, public health professionals, researchers, nurses, patients	Adapting to change, self-management, multi-sided	Functional, physical, mental, social, spiritual, others
Hunter et al., 2013 [36]	Australia	Phenomenography method	Patients and practitioners in integrative medicine clinic	Complete wellbeing or functioning, wellbeing, multi-sided	
Jormfeldt, 2009 [38]	Sweden	Cross-sectional study	Patients and staff in mental health services	Satisfying life, self-management	
Post, 2014 [45]	The Netherlands	Narrative review	NA	Functioning, subjective	Physical, mental, social, functional
Schrank et al., 2013 [46]	United Kingdom, Austria, Canada	Systematic review and narrative synthesis	People with psychosis	Daily functioning, participation, self-management, subjective	Individual
Shearer et al., 2009 [47]	USA	Qualitative descriptive design	Older women with chronic illness	Participation, satisfying life, adapting to change, self-management, subjective	
Warsop, 2009 [48]	United Kingdom	Phenomenological approach	NA	Satisfying life, daily functioning	
Zhang et al., 2014 [49]	China	Qualitative descriptive design	Chinese elderly with chronic illness, aged over 60	Multi-sided, self-management

**Table 8:** Included articles discussing health from the perspective of elderly people

Authors, year	Country	Article type/ study design	Perspective (population)	Subthemes of Concept of health	Subthemes of Dimensions of health
Boggatz, 2016 [50]	Austria	Concept analysis	Older adults	Subjective, adapting to change, satisfying life	
Cresswell-Smith et al., 2018 [51]	Finland/Italy/Norway/ Spain	Rapid review	Older adults, 80 years and older	Adapting to change, self-management, daily functioning	Functional, social, individual, environmental
Ebrahimi et al., 2012 [43]	Sweden, USA	Phenomenological approach	Elders in emergency treatment, 80 years and older, or 65 years and older with chronic diseases	Subjective, adapting to change	Individual, environmental
Fange & Ivanoff, 2009 [52]	Sweden	Grounded theory method	Old age, between 80 and 89 years old	Participation, self-management	
Goins et al., 2011 [53]	USA	Qualitative approach	community dwelling persons aged 60 years or older in west Virginia	Participation, subjective, adapting to change, satisfying life, multi-sided	Physical, functional, mental, spiritual
Noghabi et al., 2013 [54]	Iran	Theoretical analysis of literature and empirical observation. Hybrid concept analysis.	Old people, 65 years and older	Self-management	Physical, mental, social, spiritual, environmental
Shearer et al., 2009 [47]	USA	Qualitative descriptive design	Older women with chronic illness	Participation, satisfying life, adapting to change, self-management, subjective	
Song & Kong, 2015 [18]	Republic of Korea	Systematic review	Older adults	Self-management, adapting to change, satisfying life	Physical, mental, social, spiritual
Zhang et al., 2014 [49]	China	Qualitative descriptive design	Chinese elderly with chronic illness	Multi-sided, self-management

**Table 9:** Included articles discussing health from a philosophical perspective

Authors, year	Country	Article type/ study design	Perspective (theoretical approach)	Subthemes of Concept of health	Subthemes of Dimensions of health
Included articles discussing health from a social science perspective					
Bauer et al., 2020 [55]	Switzerland, Canada, Kenya, Italy, United Kingdom, Sweden, Norway, Denmark, Spain, Israel, Austria, Singapore, Netherlands,	Literature study	Salutogenic		
Bircher & Kuruvilla, 2014 [3]	Switzerland	Multi-grounded theory method	Multi-grounded theory	Wellbeing, adapting to change, multi-sided	Environmental, individual, social
Cloninger et al., 2012 [56]	USA	Literature study	Holistic	Multi-sided, adapting to change	
de Araújo et al. 2012 [57]	Brazil	Theoretical study	Hermeneutics	Subjective, adapting to change	
Elliot, 2016 [58]	United Kingdom	Literature study	Eudaimonistic	Multi-sided	
Ereshefsky, 2009 [59]	Canada	Paper	Naturalist/ normativist		Physical, mental
Haverkamp et al., 2018 [7]	The Netherlands	Practice-oriented review	Philosophical		
Huber et al. 2011 [5]	The Netherlands	Analysis	Positive health	Adapting to change, self-management	Physical, mental, social
Leonardi, 2018 [1]	Italy	Literature study	Epistemological	Self-management, adapting to change, daily functioning	
Misselbrook, 2014 [60]	Bahrain	Note	Human flourishing	Satisfying life, adapting to change	
Misselbrook, 2016 [61]	Bahrain	Literature study	Human flourishing	Satisfying life, multi-sided, adapting to change	Physical, mental, social, spiritual, others
Prinsen & Terwee, 2019 [15]	The Netherlands	Mixed-method study including a literature search, a qualitative and quantitative ranking study, followed by a content validity study	Positive health		
Reed, 2019 [62]	USA	Review	Philosophical	Subjective, satisfying life	Physical, social
Van Spijk, 2015 [63]	Switzerland	Scientific contribution	Philosophical anthropology	Satisfying life	
Sturmberg et al., 2010 [17]	Australia/USA	Literature study	Philosophical	Subjective, adapting to change, multi-sided	Physical, mental, social, functional
Sturmberg, 2014 [64]	Australia	Commentary	Philosophical	Adapting to change	
Tengland, 2016 [65]	Sweden	Critical discussion	Holistic/ capability approach	Subjective, wellbeing, multi-sided	Environmental
Tyreman, 2011 [66]	United Kingdom	Literature study	Phenomenological/ hermeneutics	Multi-sided, subjective, adapting to change, participation	
Venkatapuram, 2013 [4]	United Kingdom	Debate	Capability approach	Daily functioning, subjective, satisfying life	
Included articles discussing health from a biomedical science perspective					
Boorse, 2011 [67]	USA	Conceptual analysis	Naturalist	Complete wellbeing or functioning	
Boorse, 2014 [68]	USA	Reactions to critics	Naturalist	Complete wellbeing or functioning	
Hafen, 2016 [16]	Switzerland	Sociological systems theory	Health/health impairment-continuum	Complete wellbeing or functioning	
Schroeder, 2013 [69]	United Kingdom	Literature study	Comparative	Daily functioning

**Table 10:** Included articles discussing health from a theological perspective

Authors, year	Country	Article type/ study design	Perspective (theoretical approach or population)	Subthemes of Concept of health	Subthemes of Dimensions of health
Messer, 2013 [70]	United Kingdom	Philosophical discussion, book chapter	Theological	Satisfying life	
Proeschold-Bell et al., 2009 [71]	USA	Grounded theory approach	United Methodist church pastors	Multi-sided, satisfying life, wellbeing	Physical, mental, spiritual, others
Sadat Hoseini et al., 2015 [72]	Iran	Concept analysis	Islamic philosophy	Adapting to change, multi-sided	Physical, mental, social, spiritual
Tirodkar et al., 2011 [73]	USA	Qualitative research design	South Asian immigrants in Chicago / religion	Multi-sided	Functional, social, physical, spiritual
Walther et al., 2015 [74]	Kenya/USA	Phenomenological approach	United Methodist Church clergy	Multi-sided, wellbeing	Physical, mental, spiritual, environmental

**Table 11:** Included articles discussing health from a context specific perspective

Authors, year	Country	Article type/ study design	Perspective (population)	Subthemes of Concept of health	Subthemes of Dimensions of health
Included articles discussing health from a cultural specific perspective					
Kendall et al., 2019 [75]	Australia	Community collaborative participatory action research	Aboriginal mothers in metropolitan regional, and remote prisons	Complete wellbeing or functioning, adapting to change, self-management, multi-sided	
Mark & Lyons, 2010 [76]	New Zealand	Phenomenological approach	Māori spiritual healers	Multi-sided, satisfying life	Spiritual, environmental, others
Seyedfatemi et al., 2014 [77]	Iran	Systematic review	Iranian women’s health concepts	Multi-sided, adapting to change	Environmental, social, individual, physical, spiritual
Yang et al., 2016 [78]	Republic of Korea/USA	Qualitative method	Nepalese women, had lived in the Dadeldhura district for more than 5 years	Complete wellbeing or functioning, satisfying life, participation	
Included articles discussing health from an immigrant’s perspective					
Cha, 2013 [79]	South-Korea	Grounded theory method	Korean migrant women who migrated to North-America or Canada for their children’s education while their husbands remained in Korea	Satisfying life, daily functioning, complete wellbeing or functioning	
Martin, 2009 [80]	USA	Phenomenology	Older Iranian immigrants	Adapting to change, multi-sided	Mental, physical, spiritual, social, others
Tirodkar et al., 2011 [73]	USA	Qualitative research design	South Asian immigrants in Chicago / religion	Multi-sided	Functional, social, physical, spiritual
Included articles discussing health from an educational perspective					
Jensen, 2013 [81]	Denmark	Qualitative approach	Women with low levels of education	Wellbeing, complete wellbeing or functioning, multi-sided, satisfying life	
Stronks et al., 2018 [82]	The Netherlands	Concept mapping	Lay persons with a lower educational level	Complete wellbeing or functioning, daily functioning, multi-sided, satisfying life	
			Lay persons with an intermediate educational level	Complete wellbeing or functioning, daily functioning, multi-sided, satisfying life, self-management,	
			Lay persons with an higher educational level	Complete wellbeing or functioning, daily functioning, multi-sided, satisfying life, subjective, self-management	
Included articles discussing health from other context specific perspectives					
Mayer & Bones, 2011 [83]	Germany, South-Africa	Multi-method research	South-African managers and expatriates	Wellbeing, multi-sided, subjective	Mental, physical, spiritual
Rawolle et al., 2016 [84]	Australia	Descriptive qualitative study	South-Australian farmers	Daily functioning, participation, complete wellbeing or functioning	Individual, social, environmental

### Theme 3: concept of health from different perspectives

From the data for theme 3 (*perspective*) 68 codes were extracted and categorised. Seven subthemes arose by categorising the codes: general population (articles which do not specify a specific perspective in their study), care workers, patients, older people, philosophical, theological, and context specific (articles which define a specific context or viewpoint such as ‘Māori spiritual healers’). In the next paragraphs the similarities and differences between theme 1 (*concepts of health)* and theme 2 (*dimensions of health)* are outlined *per perspective,* in line with Tables 5, 6, 7, 8, 9, 10 and 11. We reviewed every subtheme mentioned in the included articles. We did not take into account the importance or weighting of a certain subtheme in our analyses although it was considered of higher importance in that specific article.

### Health from a general population perspective

Thirteen articles were written from a general population perspective [20–32]. These articles were mostly literature studies, discussion articles or commentaries in which health concepts were discussed. Detailed characteristics of the included articles are shown in Table 5.

In the next paragraph, illustrative quotes are given for the subthemes of theme 1 (*concept of health*) which were identified in at least three different articles. Examples of quotes are also given of associations seen between the results of theme 2 (*dimensions of health*) and theme 1 (*concept of health*). For more detailed information and all quotes see supplementary Table S2A.

Content belonging to four subthemes were identified in at least three articles written from the general population perspective: **multi-sided**, **self-management**, **participation**, and **subjective**. The subtheme **multi-sided** view on health, i.e., health not only related to the physical dimension, was identified in five articles (5/13) written from a general population perspective. For example, Amzat and Razum [21] wrote: “the concept of health presents a form of ambiguity because it is multidimensional, complex, and sometimes elusive”. The **multi-sided** view on health from this perspective was also identified by the multiple *dimensions of health* (theme 2) being reported in six articles (6/13). For example, Lipworth et al. [27] wrote: “… balance among the physical, spiritual, cognitive, emotional, and/or social domains of life”. The subtheme **self-management** as part of a health concept was identified in three articles (3/13) written from a general population perspective. For example, Makoul et al. [28] wrote about the concept of health: “Health is the result of an individual’s behaviors, and is embodied in the self-control it takes to enact the behaviors”. The subtheme **participation**, i.e., being active and participating in life, as part of a health concept was identified in three articles (3/13) written from a general population perspective. For example, Makoul et al. [28] wrote: “Health is the means to living an active life”. **Participation** as part of a health concept was also identified in the *dimension* social (theme 2). For example, Makoul et al. [28] wrote: “… the biopsychosocial model encompasses mental, emotional, social, and spiritual elements as well”. The subtheme **subjective** view on health as part of a health concept was identified in three articles (3/13) written from a general population perspective. For example, Kaldjian [25] wrote: “… we can endorse a concept of health that incorporates … subjective features of human valuing”. The other subthemes for the concepts of health were not identified in three articles or more and thus not further described here (see S2A).

### Health from a care worker’s perspective

Ten articles were written from a care workers perspective [12, 33–41]. The care workers in these articles were for example general practitioners, social workers, and staff in mental health. Characteristics of the included articles are shown in Table 6.

Content belonging to six subthemes were identified in at least three articles written from a care worker’s perspective: **multi-sided**, **subjective**, **adapting to change**, **satisfying life**, **wellbeing** and **complete wellbeing and functioning**. The subtheme **multi-sided** view on health was identified in six articles (6/10) written from a care worker’s perspective. For example, Hunter et al. [36] wrote; “health is more multidimensional” and Merry [40] wrote; “health is viewed from a holistic perspective”. The **multi-sided** view on health from this perspective was also identified by multiple *dimensions of health* (theme 2) being reported in six articles (6/10). For example, Ashcroft and Van Katwijk [34] wrote; “… health is physical, mental and emotional well-being—as determined by relationships with others and with the constructed and natural environments …”. The second subtheme, health is **subjective**, i.e., the concept of health depends on personal perceptions and experiences, was identified in four articles (4/10) written from a care worker’s perspective. For example, Merry [40] wrote; “… each person is unique and that how health is defined by a person, group, or community is subjective”. The subtheme **adapting to change**, i.e., being able to adapt to personal or environmental health-related changes and circumstances, as part of a health concept was identified in three articles (3/10) written from a care worker’s perspective. For example, Huber et al. [5] wrote; “… health as ‘the ability to adapt and to self-manage …”. The subtheme **satisfying life**, i.e., values that contribute satisfaction in life, as part of a health concept was identified in three articles (3/10) written from a care worker’s perspective. For example, Jormfeldt [38] wrote; “feeling harmony and meaningfulness in life”. The subthemes **wellbeing** and **complete wellbeing or functioning** as part of a health concept were both identified in three articles (3/10) written from the perspective ofcare workers. For example, Hunter et al. [36] wrote; “… the most advanced conception of ‘health that is more than the absence of disease’ was a liberating and expansive way of being…”. However, they also referred to health as “… health being understood only as the absence of disease”, which relates to *complete* wellbeing. Notably, the subtheme complete wellbeing or functioning was never used as a *concept of health* on its own by care workers but always in combination with other subthemes for the *concept of health*. The other subthemes for the *concepts of health* were not identified in at least three articles and are not further described here (see S2B).

### Health from a patient’s perspective

Eleven articles were written from a patient’s perspective [12, 36, 38, 42–49]. The patients in these articles were for example patients with chronic illnesses, patients in mental health services, patients with psychosis, and patients with pressure ulcers. Characteristics of the included articles are shown in Table 7.

Content belonging to six subthemes were identified in three articles or more from a patient’s perspective: **subjective**, **daily functioning**, **self-management**, **satisfying life**, **adapting to change**, and **multi-sided**. The first subtheme health as **subjective** as part of the health concept was identified in five articles (5/11) written from a patient’s perspective. For example, Post [45] wrote: “… conceptualization of health encompassed … personal evaluations of well- being” and Ebrahimi et al. [43] wrote: “… health is a subjective and dynamic phenomenon”. The **subjective view** on health from this perspective was also seen by the *dimension* individual (theme 2). For example, Schrank et al. [46] wrote: “… the domain of individual well-being represents the subjective part of the concept”. The second subtheme **daily functioning**, i.e., daily functioning in life, as part of the health concept was identified in four articles (4/11) written from a patient’s perspective. For example, Warsop [48] wrote: “Health is always in the background, letting us do what we always do” and Post [45] wrote: “… health encompassed how well people function in everyday life …”. **Daily functioning** as part of a health concept was also identified in the *dimension* functional (theme 2) by Post [45]: “Functional health, including both physical functioning in terms of self-care, mobility, and physical activity level and social role functioning in relation to family and work”. The subtheme **self-management** as part of a health concept was identified in four articles (4/11) written from a patient’s perspective. For example, Jormfeldt [38] wrote: “… to be able to manage ones daily tasks”. The subtheme **satisfying life** as part of a health concept was identified in three articles (3/11) written from a patient’s perspective. For example, Jormfeldt [38] wrote about the attitudes towards health: “… to experience meaningfulness in life…” and “… to have a peaceful and positive feeling inside…”. The subtheme **adapting to change** as part of a health concept was identified in three articles (3/11) written from a patient’s perspective. For example, Shearer et al. [47] wrote: “Health was characterized by a rhythmic pattern of living with the paradox of chronic illness; that is, constructing meanings about one’s health that enhance personal strengths while acknowledging the losses and changes brought on by their illness”. The subtheme **multi-sided view** on health was identified in three articles (3/11) written from a patient’s perspective. For example, Hunter et al. [36] wrote: “… health that is more than the absence of disease …”. The **multi-sided** view on health from this perspective was also identified by multiple *dimensions of health* (theme 2) being reported in four articles (4/11). For example, Gorecki et al. [44] wrote: “We developed a conceptual framework of HRQL [Health-Related Quality of Life] in PUs that includes four domains: PU-specific symptoms, physical functioning, psychological well-being and social functioning”. The other subthemes for the *concepts of health* were not identified in at least three articles and are not further described here (see S2C).

### Health from the perspective of elderly people

Nine articles were written from the perspective of elderly people [18, 43, 47, 49–54]. The elderly people in these articles were for example elderly people with chronic illnesses. Characteristics of the included articles are shown in Table 8.

Content belonging to five subthemes were identified in at least three articles written from the perspective of elderly people: **adapting to change**, **self-management**, **subjective**, **satisfying life**, and **participation**. The subtheme **adapting to change** as part of a health concept was identified in six articles (6/9) written from the perspective of elderly people. For example, Goins et al. [53] wrote: “… defining health as a value indicates it can be fleeting, both lost and regained” and Cresswell-Smith et al. [51] wrote about the concept of health: “… older adults have been seen to adapt and accept limitations as part of the ageing process”. The second subtheme **self-management** as part of a health concept was identified in six articles (6/9) written from the perspective of elderly people b. For example, Song and Kong [18] wrote: “… older adults experience health when they have the ability to do something independently…”. That health is **subjective** was identified in four articles (4/9) written from the perspective of elderly people. For example, Ebrahimi et al. [43] wrote: “The state of being in harmony and balance is highly individualized …”. That health is **subjective** was also identified by the *dimension* individual (theme 2). For example, Ebrahimi et al. [43] wrote: “… characterized as the individual’s experience and perception of being in harmony and balance…”. The subtheme **satisfying life** as part of a health concept was identified in four articles (4/9) written from the perspective of elderly people. For example, Song and Kong [18] wrote: “… older adults experience health when they have … connectedness with others …”. **Satisfying life** as part of a health concept was also identified in the *dimension* social and spiritual (theme 2) by Song and Kong [18]: “In addition, social, familial, and spiritual domains resonated with the theme of “connectedness with others”” [18]. The subtheme **participation** as part of a health concept was identified in four articles (4/9) written from the perspective of elderly people. For example, Fänge and Ivanoff [52] wrote: “Health was very much related to the possibility of being active and participating in social life …, and it was always evaluated in relation to their age and what they perceived could be expected in this context”. Although it was not frequently identified in the subthemes of theme 1 (*concept of health*) the **multi-sided** view on health from the perspective of elderly people was identified by multiple *dimensions of health* (theme 2) being reported in five articles (5/9). For example, Goins et al. [53] wrote: “… holistic nature of health, cut across more than 1 dimension … health cannot be compartmentalised but includes elements of physical, behavioral, psychological, and spiritual well-being”. The other subthemes for the *concepts of health* were not identified in at least three articles and are not further described here (see S2D).

### Health from a philosophical perspective

Twenty-three articles were written from a philosophical perspective. We divided the philosophical perspective articles in two groups: social science perspectives (19 articles) [1, 3–5, 7, 15, 17, 55–66] and biomedical science perspectives (4 articles) [16, 67–69]. The social science perspectives were for example holistic, phenomenological, epistemological, and philosophical anthropology (see Table 9). The biomedical science perspectives were for example naturalist and health/health impairment-continuum (see Table 9). Characteristics of the included articles are shown in Table 9.

In the articles written from a social science perspectives content belonging to four subthemes were identified in at least three articles: **adapting to change**, **multi-sided**, **subjective**, and **satisfying life**. The subtheme **adapting to change** as part of a health concept was identified in ten articles (10/19) written from a social science perspective. For example, Cloninger et al. [56] wrote about the concept of health as: “… a person as s/he adapts to an ever-changing internal and external environment”. The subtheme **multi-sided** view on health was identified in seven articles (7/19) written from a social science perspective. For example, Bircher and Kuruvilla [3] and Cloninger et al. [56] both wrote about the concept of health as: “… a complex adaptive system …”. The **multi-sided view** on health was also identified by multiple *dimensions of health* (theme 2) being reported in six articles (6/19) with a social science perspective. For example, Misselbrook [61] wrote: “But if we truly believe in a multi-sided model of health, which includes the biomedical, social, psychological, anthropological and spiritual dimensions, then we are swimming against the stream”. That health is **subjective** was identified in five articles (5/19) written from a social science perspective. For example, Sturmberg et al. [17] wrote: “The perception of being healthy is an emergent phenomenon based on individual and collective understandings of everyday realities”. The subtheme **satisfying life** as part of a health concept was identified in five articles (5/19) with a social science perspective. For example, Misselbrook [60, 61] wrote: “… health can be seen as the ability to flourish …”. In the articles from a biomedical science perspective content belonging to only one subtheme was identified in at least three articles: **complete wellbeing or functioning**. For example, Boorse [67] wrote about the concept of health as: “… each internal part to perform all its normal functions …”. The other subthemes for the *concepts of health* were not identified at least three times in the articles with a biomedical science perspective and are not further described here (see S2E).

### Health from a theological perspective

Five articles were written with a theological perspective [70–74]. The perspectives in these articles were for example United Methodist church clergy and Islamic philosophy. Characteristics of the included articles are shown in Table 10.

Content belonging to onesubtheme was identified in at least three articles: **multi-sided**. The subtheme **multi-sided** view on health was identified in four articles (4/5) written from a theological perspective. For example, Proeschold-Bell et al. [71] wrote: “… we define our final health outcome holistically to indicate that health is not merely the absence of problems but is, rather, the presence of multiple life satisfactions”. The **multi-sided** view on health from this perspective was also identified by multiple *dimensions of health* (theme 2) being reported in four articles (4/5). For example, Proeschold-Bell et al. [71] wrote: “… spiritual, emotional, physical, mental well-being”. The spiritual *dimension* was identified in a theological perspective in four articles (4/5). For example, Proeschold-Bell et al. [71] wrote: “Although spiritual well-being may not have the rigorous definition and tradition of physical and mental health, participants considered it essential …”. The other subthemes for the *concepts of health* were not identified at least three times and are not further described here (see S2F).

### Health from a context specific perspective

Eleven articles were written from a context specific perspective. We divided these articles with a context specific perspective in four groups: cultural perspectives (4 articles) [75–78], immigrant perspectives (3 articles) [73, 79, 80], educational level perspectives (2 articles) [81, 82], and other perspectives (2 articles) [83, 84] (see Table 11). These contexts are diverse and cannot be seen as one similar group. Because of heterogeneity, this subtheme was not included in supplementary Tables 3A and 3B. For characteristics of the included articles and more detailed information about these *concepts of health* related to their specific contexts see supplementary Table 2G.

## Discussion

We posited the research question whether a general health concept can guide *all* healthcare practices. It seems more likely that specific health concepts are needed for different professions or settings instead. In this scoping review, we provide an overview of articles discussing various concepts and dimensions of health, which were either general or specified to a particular context. We observed relevant differences but also similarities in the concepts and dimensions of health per context.

The variety of concepts of health already suggests that no consensus can be made on one overall concept to replace the WHO definition of health. First of all, our analysis shows that the best fitting health concept depends on the context. Besides, healthcare consumers act based on different health concepts when seeking care than care workers when providing it. This could mean that there is a misfit in the aims of healthcare consumers, compared to care workers. It is remarkable that complete wellbeing or functioning is mentioned by care workers, while healthcare consumers barely mentioned this biomedical viewpoint. Healthcare consumers value self-management, while care workers do not focus on self-management in their health concepts. Furthermore, individual health experiences can change over the course of life, due to diverse life circumstances and events [55]. It was seen that patients in general tend to focus on daily functioning while elderly people specifically focus on participation. This shows that one health concept does not automatically fit all age groups. On the other hand, there were interesting similarities regarding the concepts of health. In the majority of the articles, health was conceptualised as multi-sided and subjective, and not merely as complete wellbeing or functioning as suggested in the biomedical model. Furthermore, in the majority of the contexts other prerequisites for health were adapting to change and satisfying life. Indeed, no consensus can be made on *one* general health concept; all health concepts capture aspects that seem relevant [7].

Nevertheless, it is important to be clear about which health concept is used as a basis for development and implementations in health management, for (re)designing health policy and for research. Health concepts developed in one context do not hold automatically in other contexts. As a result, the expectations of healthcare consumers and care workers might not align in care provision. Having different understandings of the concepts of health can lead to misunderstandings in practice. Our overview of health concepts gives insight in the variety of experiences with health concepts of people with diverse health, life, community and other environmental circumstances. Policy officers or healthcare providers can check the similarities and differences of their health concept with health concepts in other contexts included in this overview. Even better, the overview we provide can be used by care workers preparing their conversation about what health means for the healthcare consumer. However, it should be emphasized that health could mean something different for each individual; no concepts are intrinsically incorrect. As Haverkamp et al. [7] described, health concepts share different features or assumptions and should be understood as a member of a family of concepts. By exploring the health concept in dialogue, important purposes of health provision can be defined by the care worker and the healthcare consumer together. Through such conversation between actors, health provision can be customised for each individual. Tools such as the positive health dialogue tool [12] might be of use in these conversations. This dialogue tool consists of six dimensions of health which correspond to the dimensions found in our study. However, the environmental dimension was not included in the positive health dialogue tool and might be of additional value to the conversation about what health means to an individual.

Many perspectives shared a similar multi-sided approach as Huber’s positive health [12]. Taking a closer look, we noticed that ‘the ability to adapt and to self-manage’, the main issues of the concept of positive health, were also recognised in other health concepts, independently of perspective. The concepts of health described the ‘ability to adapt’ for example as adapting to changing physical conditions, such as ageing, illness or disability, and also as emotional balance and as health being a dynamic state in which adaptation to circumstances is necessary. ‘The ability to self-manage’ was described for example as autonomy or independence. However, care workers had barely focussed on this. This indicates that for care workers, patient self-management has less priority. Furthermore, we noticed that subjectivity was not explicitly mentioned in Huber’s concept, while this was frequently mentioned in the articles included in our review. However, Huber et al. did explain that positive health focuses on people’s strengths rather than weaknesses. As Huber argues, people’s strengths are based on their perception of and experiences with health [12], which is subjective. Notably, as mentioned by Prinsen and Terwee [15], it is not entirely clear whether the positive health concept refers to patients’ experiences or to their satisfaction with their health, and overlap between dimensions and aspects of Positive Health exist; this was also seen in our results.

### Methodological considerations

A few methodological considerations are worth mentioning. A limitation of the search strategy was that the keyword ‘health’ by itself led to too many results. To solve this, we used the keyword ‘health’ in combination with ‘concept’ and ‘definition’ and used more specific keywords such as ‘health perception’ and ‘perceived health’ to broaden the search strategy and capture all relevlant articles for our research. Most research we found was conducted in Europe and North America. Fewer research articles from Central/South America, Australia, Africa and Asia were found. Their views on health may be underrepresented. To decrease the chance that articles were missed in the search, a snowball method was conducted on the results of the primary search. Four experts from the field were asked to check whether they missed any articles in the selection. Moreover, we did not include the weighting (importance) of a specific subtheme as was described in some articles. To compensate, we only incorporated a subtheme in our analyses by introducing a minimum level of appearance in multiple articles (> 3) as threshold. Strengths of the research were the thoroughly structured process of article selection, the inductive method of analysis, and the repeated consultation of four researchers (EB, LN-vV, EdV, DvdM) to discuss the process and the results by the first author (VvD).

## Conclusion

We performed a scoping review to explore if one general health concept can guide all different care practice situations. Based on of the variety of health concepts from different perspectives, we conclude that for every perspective, and even for every individual, health can mean something different. Thus, it seems impossible to choose or define one health concept appropriate for all contexts. However, in the interaction between care workers and healthcare consumers (and also in health policy) it is important that the meaning of ‘health’ is clear to all actors involved to avoid misunderstandings. Our overview supports a more uniform tuning of healthcare between healthcare providers (the organisations), care workers (the professionals) and healthcare consumers (the patients), by creating more awareness of the differences among these actors, which can be a guide in their communication.

## Supplementary Information

**Additional file 1: Supplementary Table 1.** The coding scheme; identified subthemes and codes for theme 1, the concept of health.

**Additional file 2: Supplementary Table 2A.** Included articles discussing health from a general population perspective. **Supplementary Table 2B.** Included articles discussing health from a care workers perspective. **Supplementary Table 2C.** Included articles discussing health from a patient’s perspective. **Supplementary Table 2D.** Included articles discussing health from the perspective of elderly people. **Supplementary Table 2E.** Included articles discussing health from a philosophical perspective. **Supplementary Table 2F.** Included articles discussing health from a theological perspective. **Supplementary Table 2G.** Included articles discussing health from a context specific perspective.

**Additional file 3: Supplementary Table 3A.** Overview of number of articles per subtheme for theme 1 (concept of health) for different perspectives. **Supplementary Table 3B.** Overview of number of articles per subtheme for theme 2 (dimensions of health) for different perspectives.

## Data Availability

The dataset (list of included articles) supporting the conclusions of this article is included within the tables in this article and in the supplementary files.
