# Supplementary material for: Concepts of health in different contexts: a scoping review
Source: BMC Health Serv Res. 2022 Mar 24;22:389. doi: 10.1186/s12913-022-07702-2 (PMC8953139; doi:10.1186/s12913-022-07702-2)
Supplement: Supplementary file 1 — Additional file 1: Supplementary Table 1. The coding scheme; identified subthemes and codes for theme 1, the concept of health. [file 12913_2022_7702_MOESM1_ESM.docx]

### Supplementary Table 1: The coding scheme; identified subthemes and codes for theme 1, the concept of health.

| **Subtheme (explanation)** | **Code** | **Quotation (example)** |
| --- | --- | --- |
| Complete wellbeing or functioning  (Functioning without any disturbance of diseases or infirmities.) | Absence of disease and functioning | *“All groups identified a cluster related to the absence of disease and disabilities with statements referring to disease, pain, discomfort, disabilities and functional abilities”.* (1) |
|  | Absence of disease or illness | *“The most prevalent definition of health was the absence of illness…”* (2) |
|  |  | *“… a state of health that is more than the absence of disease.”* (3) |
|  |  | *“… as a lack of illness.”* (4) |
|  |  | *“… absence of disease …”* (5) |
|  | Absence of health problems | *“…health was about the absence of health problems…”* (6) |
|  | Adopting the biomedical view | *“…commonly adopt the bio- medical view and equate illness and disease using the terms interchangeable.”* (7) |
|  | Biomedical interpretation of health | *“… Nevertheless, by probing deeper into their accounts, it becomes clear that this bio-medical interpretation of health is more or less entirely removed from their lives and reference frames.”* (8) |
|  | Complete physical | *“Physical. Health is completely physical. The focus is on the body and biomedical criteria (e.g., physical exams and lab tests).”* (9) |
|  | Getting off or maintaining desistance from harmful substance | *“… said that health was about the absence of health problems, overcoming health problems, and getting off or maintaining desistance from harmful substances.”* (6) |
|  | Health as a condition to be fixed | *“Many participants considered health as a condition to be fixed as the need arose, to restore functional capacity. Health from a functional perspective was seen as analogous to farm machinery.”* (10) |
|  | Health merely as absence of disease or infirmity | *“Thus, it is strongly recommended to define health merely as the absence of disease or infirmity and put the distinction of health/health impairments carefully in relation to other distinctions that describe the conditions and consequences of health without integrating them in the term health.”* (11) |
|  | No tension | *“… absence of disease, no tension, peace in the family, and being able to work...”* (5) |
|  | Normal functional ability | *“Health in a member of the reference class is normal functional ability: the readiness of each internal part to perform all its normal functions on typical occasions with at least typical efficiency.”* (12) |
|  | Normal physiological functional ability | *“…which defines health as normal physiological functional ability.”* (13) |
|  | Not getting sick | *“Their definition of health is limited to not getting sick, and continuing to be able to take care of their children.”* (14) |
|  | Theoretical health is value free | *“I claim that theoretical health, so analyzed, is value-free, since none of its component ideas requires value judgments.”* (13) |
| Wellbeing  (Wellbeing in several ways but not referring to complete wellbeing or functioning.) | Liberating and expansive way of being | *“The most advanced conception of ‘health that is more than the absence of disease’ was a liberating and expansive way of being - there were many positive attributes in all the dimensions of health that were more than just the component parts.”* (3) |
|  | Overall wellbeing | *“‘Holistic’, ‘feeling whole’, ‘health is overall wellbeing’ and ‘an overall well-being, wellness in mind, body, spirit, soul’.”* (15) |
|  | Physical-psychological wellbeing | *“All of the participants chose similar labels to define health: they labeled it both as general psycho-physical well-being and as a lack of illness…”* (4) |
|  | Positive concept of wellbeing | *“Most of the managers refer to health as a positive concept of well-being, and only a few managers view health in a ‘‘pathogenic’’ way as ‘‘absence of sickness’’.”* (16) |
|  | Sense of wellbeing | *“The participants defined health as … ‘‘a general sense of well-being’’; and ‘‘spiritual, emotional, physical, mental well-being.”* (17) |
|  |  | *“… an understanding of health being closely related to ‘the good life’ and a sense of wellbeing.”* (8) |
|  | Spiritual and emotional wellbeing | *“… defined health holistically, not merely an absence of disease or physical illness, but rather including physical health and spiritual and emotional well-being.”* (18) |
|  | State of wellbeing | *“Health is a state of wellbeing emergent from conducive interactions between individuals’ potentials, life’s demands, and social and environmental determinants.”* (19) |
|  | Subjective wellbeing | *“In conclusion, every theory of health has to take subjective wellbeing into account, in one way or another, either as a causal factor, as Nordenfelt does, or as conceptually related to health, as I have argued.”* (20) |
|  | wellbeing | *“This paper has clarified that HRQoL questionnaires describe health using functioning and well-being but this has little to do with QoL as it is known in the wider literature.”* (21) |
| Adapting to change  (Being able to adapt to personal or environmental health-related changes and  circumstances.) | Ability to adapt | *“…concept of health as ‘the ability to adapt and to self-manage, in the face of social, physical and emotional challenges’…* (22) |
|  |  | *“Resourcefulness, and the ability to adapt and deal with anything that happens is fundamental to health.”* (23) |
|  |  | *“… health, as the ability to adapt and to self manage …”* (24) |
|  | Acceptance and adjustment with optimism | *“This meta-study found that older adults experience health when they have the ability to do something independently, absence or management of symptoms, acceptance and adjustment with optimism, connectedness with others, and enough energy in their own world.”* (25) |
|  | Adapt and accept limitations as part of ageing | *“Although there is clear evidence in favour of maintaining physical capability and health in later life, older adults have been seen to adapt and accept limitations as part of the ageing process.”* (26) |
|  | Adaptation to worsening life conditions | *“In summary, quality of life in old age in terms of general well-being can be defined as a subjective state characterised … and are kept stable by adaptation to worsening life conditions.”* (27) |
|  | Adaptive system | *“The Meikirch Model of Health represents health as a complex adaptive system containing ongoing interactions between individuals’ potentials, the demands of life, and social and environmental determinants.”* (19) |
|  |  | *“… health is better conceptualized as a complex adaptive system that is dynamically self-organizing within a person as s/he adapts to an ever-changing internal and external environment, rather than a collection of independent diseases.”* (28) |
|  |  | *“Perceiving health in this way requires embracing a complex adaptive systems approach, rather than the “rational” approach of the ruling biomedical model.”* (29) |
|  | Balance among dimensions | *“It also showed that health is only feasible when balance is among these dimensions.”* (30) |
|  | Dynamic nonlinear interaction | *“The basic proposition that health is the emergent product of a dynamic nonlinear interaction between the "biological potentials", "acquire potentials", and the "demands of life" is a useful model to conceptualise the nature of health.”* (31) |
|  | Dynamic over time | *“The threshold of what constituted health was dynamic over time, and depended on the adjustment of the perceived balance point…”* (32) |
|  | Emotional balance | *“… can be defined as a subjective state characterised by the attributes life satisfaction and emotional balance which mirror the satisfaction of underlying needs and are kept stable by adaptation to worsening life conditions.”* (27) |
|  | Flow of energy, listening to and respecting its rhythms | *“Thus, health meant acknowledging this flow of energy, listening to, and respecting its rhythms as part of the meaning and manifestation of health.”* (33) |
|  | Functional adaptation | *“Health care should aim for the state of least possible illness or disability, or of maximal functional adaptation to illness or disability.”* (34) |
|  | Health and peace are dynamic | *“Health and peace are dynamic. Peace and health are not merely concerned with the absence of infirmity, war and violence.”* (35) |
|  | Health as a process | *“… conceptualized health as a process and highlighted the role of agency in a “becoming healthy process”. Becoming healthy was a sequential process with discrete steps that needed to be negotiated over time.* (6) |
|  | Health as a state of balance | *“health is presented as a state of balance and …”* (36) |
|  |  | *“… the state of balance was associated with physical or psychological health: the more one was balanced, the more likely one was to be healthy.”* (37) |
|  |  | *“… described “being health” as a state of having balance in their lives …”* (38) |
|  |  | *“… health is viewed from a holistic perspective and involves harmony or balance between body, mind, spirit and environment (i.e. wholeness).”* (39) |
|  |  | *“… health traditionally and philosophically is a personal construct based on the understanding of a balance between the different dimensions of one’s existence.”* (29) |
|  |  | *“… to achieve a harmony and balance of the essential components.”* (33) |
|  | Health can be fleeting both lost and regained | *“… defining health as a value indicates it can be fleeting, both lost and regained.”* (40) |
|  | Health is a dynamic state | *“Health is a dynamic state: it is neither solely an individual construction (illness), a reflection of societal attributes (sickness), nor a body or mind clinic-pathologic system in varying degrees of order and disorder (disease).”* (29) |
|  | Interactions | *“… the ICF conceptualization is interactional rather than linear, in the sense that disabilities are characterized as outcomes of the interaction between underlying health conditions (e.g., diseases, disorders, syndromes, and the aging process) and physical, human-built, attitudinal, and social environmental barriers.”* (41) |
|  |  | *“Health as a state of wellbeing emergent from conducive interactions between individuals’ potentials, life’s demands, and social and environmental determinants.”* (19) |
|  | Maximal functional adaptation to illness or disability | *“Health care should aim for the state of least possible illness or disability, or of maximal functional adaptation to illness or disability.”* (42) |
|  | Never-ending system of events | *“In this perspective, health may be conceived as a potentially never-ending system of events, within which an observer may select some of these events and construct a theoretical configuration of health, depending on its own theories, knowledge purposes, and operational targets…”* (43) |
|  | Overcoming health problems | *“… health was about the absence of health problems, overcoming health problems, and getting off or maintaining desistance from harmful substances.”* (6) |
|  | Process individuals go through during illness and health | *“… defined based on the process that individuals go through during illness and health.”* (44) |
|  | Rhythmic pattern of living | *“Health was characterized by a rhythmic pattern of living with the paradox of chronic illness; that is, constructing meanings about one’s health that enhance personal strengths while acknowledging the losses and changes brought on by their illness.”* (33) |
|  | Subject to change | *“Health seems to be considered as something that is, for the most part, subject to the laws of chance, and of which human beings cannot have full knowledge or complete control.”* (4) |
| Multi-sided  (Health is not related only to the physical dimension, but involves several dimensions.) | Extends beyond the physical | *“Despite the importance of physical health, a definition of health extends beyond the physical and ‘has to be more than how it is defined within a bio-medical model’.”* (15) |
|  | Health as complex system | *“The Meikirch Model of Health represents health as a complex adaptive system containing ongoing interactions between individuals’ potentials, the demands of life, and social and environmental determinants.”* (19) |
|  |  | *“Health concept is considered as multidimensional, complex, and difficult to be measured.”* (45) |
|  |  | *“… health is better conceptualized as a complex adaptive system …”* (28) |
|  |  | *“…health as a complex, multi-factorial construct, with some of the associated latent factors appearing potentially robust.”* (46) |
|  |  | *“… embracing a complex adaptive systems approach, rather than the “rational” approach of the ruling biomedical model.”* (29) |
|  | Health as comprehensive view | *“This is especially true if we think of health not just as something to be located in organs, vital signs, and bodily functions, but take a more comprehensive view that includes mental and psychological health.”* (47) |
|  | Health as holistic | *“‘holistic’, ‘feeling whole’, ‘health is overall wellbeing’ and ‘an overall well-being, wellness in mind, body, spirit, soul’.”* (15) |
|  |  | *“… define health holistically (including the mind, body, emotions, and spirit), and, … as a whole person and to not just focus on illness/disease elimination.”* (38) |
|  |  | *“… health is viewed from a holistic perspective and involves harmony or balance between body, mind, spirit, and environment (i.e., wholeness).”* (39) |
|  |  | *“… define our final health outcome holistically to indicate that health is not merely the absence of problems but is, rather, the presence of multiple life satisfactions.”* (17) |
|  |  | *“… it is notable that one third of the participants … endorsed a holistic model of health that also encompassed spiritual factors.”* (48) |
|  |  | *“In other words health is the holistic and ontological phenomenon of engagement.”* (23) |
|  |  | *“… health is holistic … defined health as more than just physical health.”* (18) |
|  |  | *“…The third theme of health’s meaning/definition, the holistic nature of health, cut across more than 1 dimension of life …”* (40) |
|  |  | *“It seems that the concept of health … through a holistic view, they all consider that the concept of health is dependent upon human consciousness and wisdom, and accordingly, emphasize superiority of spiritual health over physical health.”* (44) |
|  |  | *“… endorse a holistic theory as a general approach for defining health.”* (20) |
|  |  | *“…describing a holistic concept of health as inextricable from their culture, identity, histories, and social and structural circumstances intertwined with those of their family and community.”* (6) |
|  | Health is not merely the absence of disease or infirmity | *“…not merely an absence of disease or physical illness, but rather including physical health and spiritual and emotional well-being.”*(18) |
|  |  | *“Peace and health are not merely concerned with the absence of infirmity, war and violence.”* (35) |
|  | Health is not only normal physical function | *“Health means not only normal physical function but a responsibility for yourself and others. For my own, it lies in self-maintenance and good quality of life; if lack of quality, long-time living is meaningless.”* (49) |
|  | Mind, body, soul or spirit concept | *“Healers viewed the mind, body, soul or spirit concept as something Päkeha brought to Aotearoa/NZ…”* (50) |
|  | More than physical | *“… defined health as more than just physical health.”* (18) |
|  |  | *“… health is more than mere physical integrity …”* (29) |
|  | More than the absence of disease or illness | *“… health is more than the absence and illness more than the symptoms of disease.”* (23) |
|  |  | *“… health that is more than the absence of disease …”* (3) |
|  | Multi-facetted concept | *“… overall views about the concept of health resulted in two categories, "A multi-facetted concept" and "A subjective assessment", which are related to each other.”* (51) |
|  | Multidimensional | *“Health concept is considered as multidimensional, complex, and difficult to be measured.”* (45) |
|  |  | *“… health was more multidimensional and there was a continuum from disease to the absence of disease and then to optimum health.”* (3) |
|  |  | *“… a multidimensional model of health, which includes the biomedical, social, psycho- logical, anthropological and spiritual dimensions …”* (34) |
|  |  | *“… health personal factors include dimensions such as physical, psycho- social and spiritual. It also showed that health is only feasible when balance is among these dimensions …”* (30) |
|  |  | *“In all three groups health was conceptualised as a multidimensional concept.”* (1) |
|  |  | *“… health, regardless of the breadth of its definition, has multiple determinants …”* (52) |
|  |  | *“… health is a subjective, multidimensional construct deeply embedded in the everyday experience …”* (40) |
|  |  | *“… a more subjective and broader view of health that encompassed physical, mental, emotional, social, spiritual, financial and environmental health.”* (53) |
|  | Multidimensional, complex, elusive | *“The concept of health presents a form of ambiguity because it is multidimensional, complex, and sometimes elusive.”* (54) |
|  | Not just focus on illness/disease elimination | *“… define health holistically (including the mind, body, emotions, and spirit), and, hence, they expect their health providers to care for them as a whole person and to not just focus on illness/disease elimination.* (38) |
|  | Not merely the absence of problems | *“… define our final health outcome holistically to indicate that health is not merely the absence of problems but is, rather, the presence of multiple life satisfactions.”* (17) |
|  | Person is more than his illness | *“What most respondents generally appreciated about the new concept was that a person is described as more than his illness, and that the focus is on a person’s strength rather than his weakness.”* (22) |
|  | Salutogenic health concept | *“… health as a positive concept of well-being, and only a few … health in a ‘‘pathogenic’’ way as ‘‘absence of sickness’’. They rather refer to a ‘‘salutogenic’’ health concept that focuses on their personal and social resources. Health is interlinked with individual (life) energy and happiness rather than with work performance.”* (16) |
|  | Tied to quality of life concept | *“… is tied to the quality of life and being able to live the good life.”* (8) |
| Self-management  (Having self-control in the health process.) | Ability to do something independently | *“… experience health when they have the ability to do something independently, ….”* (25) |
|  | Ability to handle daily life activities | *“…a concept that is affected by genetic, environmental, healthcare services and lifestyle-related factors and involves proportional physical, mental, social, familial, spiritual, and economical welfare along with the ability to handle daily life activities which is measurable through medical and functional approaches.”* (55) |
|  | Ability to make health-related decisions | *“… in terms of their ability to make health-related decisions.”* (33) |
|  | Ability to self-manage | *“The first step towards using the concept of “health, as the ability to adapt and to self-manage” is to identify and characterise it for the three domains of health: physical, mental, and social. ”* (24) |
|  |  | *“… the new dynamic concept of health as ‘the ability to adapt and to self-manage, in the face of social, physical and emotional challenges’ and elaborated indicators of health …”* (22) |
|  | Absence or management of symptoms | *“… absence or management of symptoms, acceptance and adjustment with optimism, connectedness with others, and enough energy in their own world.”* (25) |
|  | Action and repetition of action in the health process | *“Becoming healthy was a sequential process with discrete steps that needed to be negotiated over time. … recognized the need for action and repetition of action in the becoming healthy process.”* (6) |
|  | Autonomy | *“Instead the framework places stronger emphasis on individual rather than societal factors, and refers to a number of specific dimensions that may be particularly relevant to people with psychosis, such as mental health and functioning, but also participation, autonomy, self-perception or self-control.”* (56) |
|  |  | *“The following domains were part of the list of the majority of the groups (i.e., three or four groups): … autonomy …”* (57) |
|  | Autonomy and independency | *“…the concept maps of the higher and inter- mediate educational groups hold a cluster on autonomy and independence.”* (1) |
|  | Being able to trust one’s ability | *“… being able to trust one’s ability.”* (58) |
|  | Capability to cope and manage malaise and wellbeing conditions | *“This newly proposed definition configures health as the capability to cope with and to manage one’s own malaise and well-being conditions.”* (43) |
|  | Control their lives | *“An alternative definition might be: health is created when individuals, families, and communities are afforded the income, education, and power to control their lives; and their needs and rights are supported by systems, environments, and policies that are enabling and conducive to better health.”* (59) |
|  | Experiencing enough energy in their own world | *“… enough energy in their own world.”* (25) |
|  | Focus on a person’s strength | *“ What most respondents generally appreciated about the new concept was that a person is described as more than his illness, and that the focus is on a person’s strength rather than his weakness.”* (22) |
|  | Independence | *The following domains were part of the list of the majority of the groups (i.e., three or four groups):”… independence …”* (57) |
|  | Manage daily activities | *“Health was described in terms of being able to manage daily activities at home and to participate in society.”* (60) |
|  | Manage ones daily tasks | *“…to be able to manage ones daily tasks’’…* (58) |
|  | Positive thinking and resourcefulness | *“Positive thinking and resourcefulness have been identified as mediating challenges, reflected in a tendency towards defining health in relation to abilities, not in terms of symptoms and disease.”* (26) |
|  | Responsibility for yourself and others | *“… ‘Health means not only normal physical function but a responsibility for yourself and others.”* (49) |
|  | Self-acceptance | *“The results showed that all five groups agreed on few domains. That is, only ‘self-acceptance’ is part of the highest mean ratings of all groups...”* (57) |
|  | Self-control | *“Health is the result of an individual’s behaviors, and is embodied in the self-control it takes to enact the behaviors.”* (9) |
|  |  | *“Instead the framework places stronger emphasis on individual rather than societal factors, and refers to a number of specific dimensions that may be particularly relevant to people with psychosis, such as mental health and functioning, but also participation, autonomy, self-perception or self-control.”* (56) |
|  | Self-esteem | *“… When looking at the list of five most important domains, ‘self-esteem’ and ‘good social contacts’ are the only two domains on which all five groups agree that they are highly important.”* (57) |
|  | Self-esteem, self-concept | *“Psychosocial. The focus here is on the mental, emotional, spiritual, and social aspects of health, including self-esteem and self-concept.”* (9) |
|  | To be aware of ones worth | *“… to be aware of ones worth…”* (58) |
|  | To feel secure in oneself | *“…to feel secure in oneself…”* (58) |
| Participation  (Being active and participating in life.) | Ability to be active and participating | *“Health was very much related to the possibility of being active and participating in social life in keeping with one’s own preferences, and it was always evaluated in relation to their age and what they perceived could be expected in this context.”* (60) |
|  |  | *“Women defined health consistent with the extent to which they were able to remain active and care for others through their contributions to family and society.”* (33) |
|  | Ability to live an active life | *“Health is the means to living an active life. It is an enabling factor. The theme here is ‘‘ability.’’”* (9) |
|  | Being able to perform activities of daily living | *“The following domains were part of the list of the majority of the groups (i.e., three or four groups): “… being able to perform activities of daily living that are …”* (57) |
|  | Being able to work | *“… absence of disease, no tension, peace in the family, and being able to work.”* (5) |
|  | Capacity to perform tasks and fulfil societal roles | *“Health can be conceptualized as the capacity to perform certain tasks and fulfil societal roles.”* (40) |
|  | Dynamic participation in the world | *“Health is the retentir that emanates from being, from our dynamic participation in the World.”* (23) |
|  | Health as basic necessity or requirements to engage in activities | *“Rather, health was described as the basic necessity or requirements to engage in all other activities.”* (53) |
|  | Participating in daily life | *“At the individual level, participants described health as the capacity to function and participate fully in daily life.”* (10) |
|  | Participation | *“Instead the framework places stronger emphasis on individual rather than societal factors, … mental health and functioning, but also participation, autonomy, self-perception or self-control.”* (56) |
| Satisfying life  (Values that contribute satisfaction in life.) | Ability to flourish | *“Thus, health can be seen as the ability to flourish without being unduly impeded by illness or disability or, if necessary, by overcoming illness or disability.”* (34) |
|  |  | *Thus, health can be seen as the ability to flourish without being unduly impeded by illness or disability or, if necessary, by overcoming illness or disability.”* (42) |
|  | Ability to live a life that makes sense | *“…Human health—also called ‘great health’—is the ability to live a life that makes sense.”* (61) |
|  | Ability to satisfy by themselves the needs of daily live | *“The health is physical and mental efficiency, it is the ability to satisfy by themselves the needs of daily life”.”* (4) |
|  | Ability to take care of children | *“… conceptualized health in relation to their newly defined selves as mothers. Being healthy meant being able to take care of their children…”* (14) |
|  | Attitude towards life | *“… also one’s attitude towards life was considered to be an aspect of health.”* (1) |
|  | Being in the world | *“If we think about health in this, phenomenological, way, we can see that it is not being viewed as a property inhering in human subjects but refers to how we are Being-in-the-world.”* (62) |
|  | Capacity to realize creaturely flourishing | *“If human creaturely flourishing is understood in theological terms, then it seems promising to conceptualize health as the capacity to realize (some aspects of) that creaturely flourishing.”* (63) |
|  | Caring for others | *“… defined health consistent with the extent to which they were able to remain active and care for others through their contributions to family and society.”* (33) |
|  | Connectedness with others | *“…experience health when they have the ability to do something independently, absence or management of symptoms, acceptance and adjustment with optimism, connectedness with others, and enough energy in their own world.”* (25) |
|  | Contextual features of human society | *“… then we can endorse a concept of health that incorporates objective features of human biology, subjective features of human valuing, and contextual features of human society.”* (64) |
|  | Experience harmony in life | *“… to experience harmony in life …”* (58) |
|  | Experience meaningfulness in life | *“… to experience meaningfulness in life …”* (58) |
|  |  | *“… health is regarded as something real about which there are facts that are meaningful in everyday life …”* (65) |
|  | Feel hope for the future | *“… to feel hope for the future …”* (58) |
|  | Good social contacts | *“… ‘self-esteem’ and ‘good social contacts’ are the only two domains on which all five groups agree that they are highly important.”* (57) |
|  | Have a peaceful and positive feeling inside | *“… to have a peaceful and positive feeling inside …”* (58) |
|  | Health as a commodity | *“Our participants had a shared meaning of health as a value or commodity.”* (40) |
|  | Health as a value | *“One of the more dominant findings was the degree to which participants placed value on their health.”* (40) |
|  |  | *“Our participants had a shared meaning of health as a value or commodity.”* (40) |
|  | Health is about the whole life | *“"Health is about life, the whole life" became the third category for the health professional's conceptual framing of health.”* (51) |
|  | Life satisfaction | *“… in terms of general well-being can be defined as a subjective state characterised by the attributes life satisfaction and emotional balance which mirror the satisfaction of underlying needs and are kept stable by adaptation to worsening life conditions.”* (27) |
|  | Life worthy of equal human dignity | *“… the capability to achieve a cluster of basic capabilities to be and do things that reflect a life worthy of equal human dignity.”* (66) |
|  | Optimism | *The following domains were part of the list of the majority of the groups (i.e., three or four groups): “… optimism …”* (57) |
|  | Peace in the family | *“… absence of disease, no tension, peace in the family, and being able to work.”* (5) |
|  | Presence of multiple life satisfactions | *“In this model, we define our final health outcome holistically to indicate that health is not merely the absence of problems but is, rather, the presence of multiple life satisfactions.”* (17) |
|  | Purpose in life | *The following domains were part of the list of the majority of the groups (i.e., three or four groups): “… purpose in life …”* (57) |
|  | Relationships with family | *“… the external relationships people have with their family/genealogy and with the land are viewed as just as important for maintaining good health.* (50) |
|  | Social life satisfactory | *“… all groups also conceptualised health in terms of having a satisfactory social life.”* (1) |
|  | Suffering as natural part of life | *“… to see suffering as a natural part of life...”* (58) |
|  | To live the good life | *“Evidently, the interview accounts demonstrate that health … is tied to the quality of life and being able to live the good life.”* (8) |
|  | Understanding of the goods, goals, and ends of human life | *“… conceptualizing health that locates it within an understanding of the goods, goals, and ends of human creaturely life.”* (63) |
| Subjective  (Personal perceptions and experiences about health.) | Bodily phenomena | *“… the experience of disability is inextricably linked to underlying impairments, which are bodily phenomena.”* (41) |
|  | Current feelings | *“… when they were assessing their health they relied on their current feelings, not on the knowledge about their current health state or the possible divergence between the actual and desired state of health.”* (67) |
|  |  | *“… the concept of health … has to do with how they feel and what they are able to do.”* (20) |
|  | Disability is a state or experience of individuals | *“… disability is a state or experience of individuals, associated with their bodies and how their bodies function that is often a disadvantage or problem that interferes with their lives and life plans.”* (41) |
|  | Enhancing personal strength | *“… constructing meanings about one’s health that enhance personal strengths while acknowledging the losses and changes brought on by their illness.”* (33) |
|  | Existential and subjective perspective of human experience | *“On the contrary, health is linked to the individual being, in an existential and subjective perspective of human experience in the face of the world of life…”* (36) |
|  | Experience of the being | *“Health is presented as a state of balance and, as such, something based on the experience of the ‘being’ in the face of the world of life, which is his/her everyday life, constructed from which elements are brought by culture, inter-subjectivity and language.”* (36) |
|  | Health as a resource for daily living | *“…health as a resource for daily living.”* (66) |
|  | Health beliefs | *“But the deeper confusion is to assume that a person’s health state logically depends on their beliefs about what health is.”* (41) |
|  | Health is based on individual and collective understandings of everyday realities | *“Instead, the perception of being healthy is an emergent phenome- non based on individual and collective understandings of everyday realities.”* (29) |
|  | Health is subjective | *“… each person is unique and that how health is defined by a person, group, or community is subjective.”* (39) |
|  |  | *“… a more subjective and broader view of health that encompassed physical, mental, emotional, social, spiritual, financial and environmental health.”* (53) |
|  |  | *“… Our results expand on previous studies and demonstrate that health is a subjective, multidimensional construct deeply embedded in the everyday experience …”* (40) |
|  |  | *“The findings from this study emphasize that health is a subjective and dynamic phenomenon.”* (32) |
|  |  | *“They consider health as a subjective experience or feeling rather than an objective confirmable state.”* (51) |
|  | Perceived health | *“.. items on self-perceived health (eg, being vital) are also included in this cluster, besides a separate cluster on ‘perceived health’, which is located close to the cluster on functioning and absence of disease.”* (1) |
|  | Personal and social resources | *“They rather refer to a ‘‘salutogenic’’ health concept that focuses on their personal and social resources.”* (16) |
|  | person-centered and society-centered perspectives and values | *“The philosophical perspec- tive of this model of health is a form of naturalism that is stance-dependent; it incorporates relevant person-centered and society-centered perspectives and values into conceptual understandings of health and practical understandings of how health occurs and may be facilitated.”* (65) |
|  | Personal evaluation of wellbeing | *“… the emerging conceptualization of health encompassed how well people function in everyday life and personal evaluations of well- being.”* (68) |
|  | Personal experience | *“We thus propose a complex adaptive systems metaphor for understanding the interdependent nature of health, health care, and the health-care system, built around the notion of health as a personal experience.”* (29) |
|  | Phenomenological ontology | *“On this account, health is more than a biological or psychological state, it is a phenomenological ontology that is experienced through our relationship with the world around us.”* (23) |
|  | Self-perception | *“Instead the framework places stronger emphasis on individual rather than societal factors, … such as mental health and functioning, but also participation, autonomy, self-perception or self-control.”* (56) |
|  | Subjective experience | *“Commonly shared attributes of health inherent in all of these definitions, however, is that it is a subjective experience that encompasses how a person is feeling and doing.”* (7) |
|  | Subjective features of human valuing | *“… then we can endorse a concept of health that incorporates objective features of human biology, subjective features of human valuing, and contextual features of human society.”* (64) |
|  | Subjective state | *“… quality of life in old age in terms of general well-being can be defined as a subjective state characterised by the attributes life satisfaction and emotional balance....”* (27) |
|  | Subjective wellbeing | *“In conclusion, every theory of health has to take subjective wellbeing into account, in one way or another, either as a causal factor, as Nordenfelt does, or as conceptually related to health, as I have argued.”* (20) |
| Daily functioning  (Daily functioning in life.) | Ability to achieve a basic cluster of beings and doings | *“… the health of an individual should be understood as the ability to achieve a basic cluster of beings and doings – or having the overarching capability, a meta-capability, to achieve a set of basic inter-related capabilities and functioning’s.”* (69) |
|  | Avoiding undesirable responses | *“… health may be conceptualized as the capability to react to all kinds of environmental events having the desired emotional, cognitive, and behavioral responses and avoiding those undesirable ones.”* (43) |
|  | Do what we always do | *“Health is always in the background, letting us do what we always do.”* (62) |
|  | Functional health | *“… agreed the most with functional health, which focused on role performance in the Health Conception Scale, followed by adaptive health, clinical health, and eudaemonistic health.”* (14) |
|  | Functional states | *“It is my contention that the concept of functional states (described in other literature as “basic functions,” “basic capabilities,” or “basic abilities”) is best understood not scientifically, but metaphysically.”* (41) |
|  | Functionalist | *“A large majority of the theories of health on offer could be described as broadly functionalist in the following respect: they declare an organism healthy based on whether the organism (or some part of the organism) can do something.”* (70) |
|  | Functionality and ability | *“These findings underline the need to support functional ability despite the existence of ill health, encouraging participation, and defining wellbeing via functionality and ability rather than on impairments.”* (26) |
|  | Functioning | *“This paper has clarified that HRQoL questionnaires describe health using functioning and well-being but this has little to do with QoL as it is known in the wider literature.”* (21) |
|  |  | *“… perceive health as being able to function and work on the farm.”* (10) |
|  | Functioning in everyday life | *“… the emerging conceptualization of health encompassed how well people function in everyday life and personal evaluations of well- being.”* (68) |
|  | Having desired emotional, cognitive, behavioural responses | *“In more operative terms, health may be conceptualized as the capability to react to all kinds of environmental events having the desired emotional, cognitive, and behavioral responses and avoiding those undesirable ones.”* (43) |
|  | Health-related behaviour | *“Clusters related to health-related behaviour were present in all groups.”* (1) |
|  | Mental health and functioning | *“Instead the framework places stronger emphasis on individual rather than societal factors, … such as mental health and functioning, but also participation, autonomy, self-perception or self-control.”* (56) |
|  | Objective features of human biology | *“… then we can endorse a concept of health that incorporates objective features of human biology, subjective features of human valuing, and contextual features of human society.”* (64) |

## References

1. Stronks K, Hoeymans N, Haverkamp B, den Hertog FRJ, van Bon-Martens MJH, Galenkamp H, et al. Do conceptualisations of health differ across social strata? A concept mapping study among lay people. BMJ Open. 2018;8(4):e020210.

2. Conner AL, Boles DZ, Markus HR, Eberhardt JL, Crum AJ. Americans’ Health Mindsets: Content, Cultural Patterning, and Associations With Physical and Mental Health. Ann Behav Med. 2019;53(4):321–32.

3. Hunter J, Marshall J, Corcoran K, Leeder S, Phelps K. A positive concept of health – Interviews with patients and practitioners in an integrative medicine clinic. Complement Ther Clin Pract. 2013;19(4):197–203.

4. Pace CS, Velotti P, Zavattini GC. Representations of health and illness by Eastern European, South American and Italian care workers: a qualitative study. J Health Psychol. 2011;17(4):490–9.

5. Yang Y, Bekemeier B, Choi J. A cultural and contextual analysis of health concepts and needs of women in a rural district of Nepal. Glob Health Promot. 2016;25(1):15–22.

6. Kendall S, Lighton S, Sherwood J, Baldry E, Sullivan E. Holistic Conceptualizations of Health by Incarcerated Aboriginal Women in New South Wales, Australia. Qual Health Res. 2019;29(11):1549–65.

7. Lyon BL. Stress, Coping, and Health, a conceptual overview. In: Handbook of stress, coping, and health: Implications for nursing research, theory, and practice. Sage Publications, Inc; 2012. p. 2–20.

8. Jensen JM. Everyday life and health concepts among blue-collar female workers in Denmark: implications for health promotion aiming at reducing health inequalities. Glob Health Promot. 2013;20(2):13–21.

9. Makoul G, Clayman ML, Lynch EB, Thompson JA. Four Concepts of Health in America: Results of National Surveys. J Health Commun. 2009;14(1):3–14.

10. Rawolle TA, Sadauskas D, van Kessel G, Dollman J. Farmers’ perceptions of health in the Riverland region of South Australia: ‘If it’s broke, fix it’’’. Aust J Rural Health. 2016;24(5):312–6.

11. Hafen M. Of what use (or harm) is a positive health definition? J Public Health (Bangkok). 2016;24(5):437–41.

12. Boorse C. Concepts of Health and Disease. 2011;13–64.

13. Boorse C. A Second Rebuttal On Health. J Med Philos. 2014;39(6):683–724.

14. Cha C. Health Concept and Health Promotion Process Among Korean Migrant Women. Health Care Women Int. 2013;34(8):628–50.

15. Ashcroft R, Van Katwyk T. Joining the Global Conversation: Social Workers Define Health Using a Participatory Action Research Approach. Br J Soc Work. 2016;bcw005.

16. Mayer C-H, Boness C. Concepts of health and well-being in managers: An organizational study. Int J Qual Stud Health Well-being. 2011;6(4):7143.

17. Proeschold-Bell RJ, LeGrand S, James J, Wallace A, Adams C, Toole D. A Theoretical Model of the Holistic Health of United Methodist Clergy. J Relig Health. 2009;50(3):700–20.

18. Walther NG, Proeschold-Bell RJ, Benjamin-Neelon S, Adipo S, Kamaara E. “We Hide Under the Scriptures”: Conceptualization of Health Among United Methodist Church Clergy in Kenya. J Relig Health. 2014;54(6):2235–48.

19. Bircher J, Kuruvilla S. Defining health by addressing individual, social, and environmental determinants: New opportunities for health care and public health. J Public Health Policy. 2014;35(3):363–86.

20. Tengland P-A. Venkatapuram’s Capability theory of Health: A Critical Discussion. Bioethics. 2016;30(1):8–18.

21. Karimi M, Brazier J. Health, Health-Related Quality of Life, and Quality of Life: What is the Difference? Pharmacoeconomics. 2016;34(7):645–9.

22. Huber M, van Vliet M, Giezenberg M, Winkens B, Heerkens Y, Dagnelie PC, et al. Towards a patient-centred operationalisation of the new dynamic concept of health. Br Med J open. 2016;6(1):1–12.

23. Tyreman S. The happy genius of my household: phenomenological and poetic journeys into health and illness. Med Heal Care Philos. 2011;14(3):301–11.

24. Huber M, Knottnerus JA, Green L, Horst H v d, Jadad AR, Kromhout D, et al. How should we define health? Bmj. 2011;343(jul26 2):d4163–d4163.

25. Song M, Kong E-H. Older adults’ definitions of health: A metasynthesis. Int J Nurs Stud. 2015;52(6):1097–106.

26. Cresswell-Smith J, Amaddeo F, Donisi V, Forsman AK, Kalseth J, Martin-Maria N, et al. Determinants of multidimensional mental wellbeing in the oldest old: a rapid review. Soc Psychiatry Psychiatr Epidemiol. 2018;54(2):135–44.

27. Boggatz T. Quality of life in old age - a concept analysis. Int J Older People Nurs. 2016;11(1):55–69.

28. Cloninger R, Salloum IM, Mezzich JE. The dynamic origins of positive health and wellbeing. Int J Pers Cent Med. 2012;2(2):179–87.

29. Sturmberg JP, Martin CM, Moes MM. Health at the Center of Health Systems Reform: How Philosophy Can Inform Policy. Perspect Biol Med. 2010;53(3):341–56.

30. Seyedfatemi N, Salsali M, Rezaee N, Rahnavard Z. Women’s Health Concept, A Meta-Synthesis Study. Iran J Public Health. 2014;43(10):1335–44.

31. Sturmberg JP. Emergent properties define the subjective nature of health and disease. J Public Health Policy. 2014;35(3):414–9.

32. Ebrahimi Z, Wilhelmson K, Moore CD, Jakobsson A. Frail Elders’ Experiences With and Perceptions of Health. Qual Health Res. 2012;22(11):1513–23.

33. Shearer NBC, Fleury JD, Reed PG. The Rhythm of Health in Older Women With Chronic Illness. Res Theory Nurs Pract. 2009;23(2):148–60.

34. Misselbrook D. W is for Wellbeing and the WHO definition of health. Br J Gen Pract. 2014;64(628):582.

35. Abuelaish I, Goodstadt MS, Mouhaffel R. Interdependence between health and peace: a call for a new paradigm. Health Promot Int. 2020;1–11.

36. de Araújo JL, Araujo Paz EP, Moreira TMM. Hermeneutics and health, reflections on the thinking of Hans-Georg Gadamer. Rev da Esc Enferm da USP. 2012;46(1):194–201.

37. Lipworth WL, Hooker C, Carter SM. Balance, Balancing, and Health. Qual Health Res. 2011;21(5):714–25.

38. Martin SS. Healthcare-Seeking Behaviors of Older Iranian Immigrants: Health Perceptions and Definitions. J Evid Based Soc Work. 2009;6(1):58–78.

39. Merry L. Global health for nursing...and nursing for global health. Can J Nurs Res. 2012;44(4):20–35.

40. Goins RT, Spencer SM, Williams K. Lay Meanings of Health Among Rural Older Adults in Appalachia. J Rural Heal. 2011;27(1):13–20.

41. Bickenbach J. Being unhealthy, and rights to health. J law, Med ethics. 2013;41(4):821–8.

42. Misselbrook D. Aristotle, Hume and the goals of medicine. J Eval Clin Pract. 2016;22(4):544–9.

43. Leonardi F. The Definition of Health: Towards New Perspectives. Int J Heal Serv. 2018;48(4):735–48.

44. Sadat Hoseini AS, Khosro Panah AH, Alhani F. The Concept Analysis of Health Based on islamic sources, intellectual health. Int J Nurs Knowl. 2015;26(3):113–20.

45. Alslman ET, Ahmad MM, Bani Hani MA, Atiyeh HM. Health: A Developing Concept in Nursing. Int J Nurs Knowl. 2017;28(2):64–9.

46. Downey CA, Chang EC. Assessment of everyday beliefs about health: The Lay Concepts of Health Inventory, college student version. Psychol Health. 2013;28(7):818–32.

47. Elliot D. Defining the Relationship Between Health and Well-being in Bioethics. New Bioeth. 2016;22(1):4–17.

48. Tirodkar MA, Baker DW, Makoul GT, Khurana N, Paracha MW, Kandula NR. Explanatory Models of Health and Disease Among South Asian Immigrants in Chicago. J Immigr Minor Heal. 2010;13(2):385–94.

49. Zhang H, Shan W, Jiang A. The meaning of life and health experience for the Chinese elderly with chronic illness: A qualitative study from positive health philosophy. Int J Nurs Pract. 2014;20(5):530–9.

50. Mark GT, Lyons AC. Maori healers’ views on wellbeing: The importance of mind, body, spirit, family and land. Soc Sci Med. 2010;70(11):1756–64.

51. Johansson H, Weinehall L, Emmelin M. ‘It depends on what you mean’: a qualitative study of Swedish health professionals’ views on health and health promotion. BMC Health Serv Res. 2009;9(1).

52. Frenk J, Gómez-Dantés O. Designing a framework for the concept of health. J Public Health Policy. 2014;35(3):401–6.

53. Thumboo J, Ow MYL, Uy EJB, Xin X, Chan ZYC, Sung SC, et al. Developing a comprehensive, culturally sensitive conceptual framework of health domains in Singapore. PLoS One. 2018;13(6):e0199881.

54. Amzat J, Razum O. Health, Disease, and Illness as Conceptual Tools. In: Medical sociology in Africa. 2014. p. 21–37.

55. Noghabi AA, Alhani F, Peyrovi H. Health Hybrid Concept Analysis in Old People. Glob J Health Sci. 2013;5(6).

56. Schrank B, Bird V, Tylee A, Coggins T, Rashid T, Slade M. Conceptualising and measuring the well-being of people with psychosis: Systematic review and narrative synthesis. Soc Sci Med. 2013;92:9–21.

57. Pietersma S, de Vries M, van den Akker-van Marle ME. Domains of quality of life: results of a three-stage Delphi consensus procedure among patients, family of patients, clinicians, scientists and the general public. Qual Life Res. 2013;

58. Jormfeldt H. Attitudes towards health among patients and staff in mental health services: a comparison of ratings of importance of different items of health. Soc Psychiatry Psychiatr Epidemiol. 2009;45(2):225–31.

59. Shilton T, Sparks M, McQueen D, Lamarre MC, Jackson S. Proposal for new definition of health. Bmj. 2011;343(aug23 4):d5359–d5359.

60. Fänge A, Ivanoff SD. The home is the hub of health in very old age: Findings from the ENABLE-AGE Project. Arch Gerontol Geriatr. 2009;48(3):340–5.

61. van Spijk P. On human health. Med Heal Care Philos. 2014;18(2):245–51.

62. Warsop A. Medically unexplained symptoms and the meaning of health, a phenomenological clue. Psychiatry. 2009;8(5):149–52.

63. Messer N. Philosophical Accounts of Health, Disease, and Illness. In: Flourishing : Health, disease, and bioethics in theological perspective. William B. Eerdmans publishing company; 2013. p. 1–50.

64. Kaldjian LC. Concepts of health, ethics, and communication in shared decision making. Commun Med. 2017;14(1):83–95.

65. Reed PG. Adaptive Preferences: A Philosophical Issue Raised by an Expanded Model of Health. Nurs Sci Q. 2019;32(3):201–6.

66. Williamson DL, Carr J. Health as a resource for everyday life: advancing the conceptualization. Crit Public Health. 2009;19(1):107–22.

67. Bąk-Sosnowska M, Skrzypulec-Plinta V. Health behaviors, health definitions, sense of coherence, and general practitioners’ attitudes towards obesity and diagnosing obesity in patients. Arch Med Sci. 2017;2:433–40.

68. Post M. Definitions of Quality of Life: What Has Happened and How to Move On. Top Spinal Cord Inj Rehabil. 2014;20(3):167–80.

69. Venkatapuram S. Health, Vital Goals, and Central Human Capabilities. Bioethics. 2013;27(5):271–9.

70. Schroeder SA. Rethinking Health: Healthy or Healthier than? Br J Philos Sci. 2012;64(1):131–59.
