# Supplementary material for: Concepts of health in different contexts: a scoping review
Source: BMC Health Serv Res. 2022 Mar 24;22:389. doi: 10.1186/s12913-022-07702-2 (PMC8953139; doi:10.1186/s12913-022-07702-2)
Supplement: Supplementary file 2 — Additional file 2: Supplementary Table 2A. Included articles discussing health from a general population perspective. Supplementary Table 2B. Included articles discussing health from a care workers perspective. Supplementary Table 2C. Included articles discussing health from a patient’s perspective. Supplementary Table 2D. Included articles discussing health from the perspective of elderly people. Supplementary Table 2E. Included articles discussing health from a philosophical perspective. Supplementary Table 2F. Included articles discussing health from a theological perspective. Supplementary Table 2G. Included articles discussing health from a context specific perspective. [file 12913_2022_7702_MOESM2_ESM.docx]

### Supplementary table 2A: Included articles discussing health from a general population perspective

| **Authors, year** | **Country** | **Article type/ study design** | **Perspective (population)** | **Theme Concept of health** | **Subthemes of Concept of health** | **Subthemes of Dimensions of health** |
| --- | --- | --- | --- | --- | --- | --- |
| Abuelaish et al., 2020 (1) | Canada | Literature debate | NA | *“Health and peace are dynamic. Peace and health are not merely concerned with the absence of infirmity, war and violence.”* | Multi-sided, adapting to change | Social, environmental |
| Amzat & Razum, 2014 (2) | Nigeria | Book chapter | NA | *“The concept of health presents a form of ambiguity because it is multidimensional, complex, and sometimes elusive.”* | Multi-sided |  |
| Conner et al., 2019 (3) | USA | Survey research | African American, Asian American, European American, and Latin American men and women of lower and higher socioeconomic status (SES) | *“The most prevalent definition of health was the absence of illness, and the number of health definition, cause, and benefit themes participants mentioned correlated with poor health outcomes. In other words, participants’ health mindsets were associated with illness, not health.”* | Complete wellbeing or functioning | Functional, physical, mental, social, spiritual, others |
| Downey & Chang 2013 (4) | USA | Empirical mixed-method study | American adults | *“… health as a complex, multi-factorial construct, with some of the associated latent factors appearing potentially robust.”* | Multi-sided |  |
| Frenk & Gómez-Dantés, 2014 (5) | USA, Mexico | Commentary | NA | *“… health, regardless of the breadth of its definition, has multiple determinants.”* | Multi-sided |  |
| Kaldjian, 2017 (6) | USA | Forum discussion | NA | *“If we can agree that shifting currents in the history of medicine reveal a malleability in the concept of disease (and therefore notions of health), and if we can also grant that health has an objective aspect that is independent of the subjective interpretations of individual patients and cultures, then we can endorse a concept of health that incorporates objective features of human biology, subjective features of human valuing, and contextual features of human society.”* | Daily functioning, subjective, satisfying life |  |
| Karimi & Brazier, 2016 (7) | Switzerland | Current opinion | NA | *“This paper has clarified that HRQoL questionnaires describe health using functioning and well-being but this has little to do with QoL as it is known in the wider literature.”* | Daily functioning, wellbeing |  |
| Lipworth et al., 2011 (8) | Australia | Qualitative literature review | NA | *“In most cases, the state of balance was associated with physical or psychological health: the more one was balanced, the more likely one was to be healthy. Indeed, in some cases, people saw the state of balance as the definition of health. This was particularly common in non-Western populations, for whom health was defined as balance among the physical, spiritual, cognitive, emotional, and/or social domains of life.”* | Adapting to change | Physical, spiritual, mental, social |
| Makoul et al., 2009 (9) | USA | Survey research | American adults | *“Capacity. Health is the means to living an active life. It is an enabling factor. The theme here is ‘‘ability.’’ Control. Health is the result of an individual’s behaviors, and is embodied in the self-control it takes to enact the behaviors. Physical. Health is completely physical. The focus is on the body and biomedical criteria (e.g., physical exams and lab tests). Psychosocial. The focus here is on the mental, emotional, spiritual, and social aspects of health, including self-esteem and self-concept.”* | Participation, self-management, complete wellbeing or functioning | Physical, mental, social, spiritual, functional, others |
| Pietersma et al, 2014 (10) | The Netherlands | Three-stage Delphi-procedure | Patients, family members of patients, clinicians, scientific experts, and general population | *“The results showed that all five groups agreed on few domains. That is, only ‘self-acceptance’ is part of the highest mean ratings of all groups. When looking at the list of five most important domains, ‘self-esteem’ and ‘good social contacts’ are the only two domains on which all five groups agree that they are highly important.”* | Self-management, satisfying life, participation | Mental, social, physical |
| Shilton et al., 2011 (11) | Australia, France | Letter to the editor | NA | *“An alternative definition might be: health is created when individuals, families, and communities are afforded the income, education, and power to control their lives; and their needs and rights are supported by systems, environments, and policies that are enabling and conducive to better health.”* | Self-management |  |
| Thumboo et al., 2018 (12) | Singapore, Finland | Qualitative research design | General public in Singapore | *“English-speaking participants generally gave a more subjective and broader view of health that encompassed physical, mental, emotional, social, spiritual, financial and environmental health. Finances and work were not cited as important aspects related to health or QoL. Rather, health was described as the basic necessity or requirements to engage in all other activities.”* | Subjective, participation, multi-sided | Physical, mental, social, spiritual, environmental |
| Williamson et al., 2009 (13) | Canada | Literature study | NA | *“At the very least, regardless of whether public health practitioners, policy makers, and scholars determine that a capital resource definition of health advances their efforts to enhance health, it is important to understand what it means to think about and treat health as a resource for daily living.”* | Subjective |  |

### Supplementary table 2B: Included articles discussing health from a care workers perspective

| **Authors, year** | **Country** | **Article type/ study design** | **Perspective (population)** | **Theme Concept of health** | **Subthemes of Concept of health** | **Subthemes of Dimensions of health** |
| --- | --- | --- | --- | --- | --- | --- |
| Alslman et al., 2015 (14) | Jordan | Concept analysis | NA | *“Health concept is considered as multidimensional, complex, and difficult to be measured.”* | Multi-sided | Physical, mental, social |
| Ashcroft & van Katwyk, 2016 (15) | Canada | Participatory action research | Social work educators, practitioners and students | *“Holistic’, ‘feeling whole’, ‘health is overall wellbeing’ and ‘an overall well-being, wellness in mind, body, spirit, soul’… Despite the importance of physical health, a definition of health extends beyond the physical and ‘has to be more than how it is defined within a bio-medical model’.”* | Multi-sided, wellbeing | Mental, physical, social, spiritual, environmental |
| Bąk-Sosnowska et al., 2017 (16) | Poland | Survey research | General practitioners | *“The examined doctors defined health most often as a state and least often as a target. This means that when they were assessing their health they relied on their current feelings, not on the knowledge about their current health state or the possible divergence between the actual and desired state of health.”* | Subjective |  |
| Huber et al., 2016 (17) | The Netherlands | Mixed method study, qualitative approach, quantitative approach | Physicians, physiotherapists, policymakers, insurers, public health professionals, researchers, nurses, patients | *“… the new dynamic concept of health as ‘the ability to adapt and to self-manage, in the face of social, physical and emotional challenges’ and elaborated indicators of health … What most respondents generally appreciated about the new concept was that a person is described as more than his illness, and that the focus is on a person’s strength rather than his weakness.”* | Adapting to change, self-management, multi-sided | Functional, physical, mental, social, spiritual, others |
| Hunter et al., 2013 (18) | Australia | Phenomenography method | Patients and practitioners in integrative medicine clinic | *“The simplest conceptions were narrow, limited to physical and mental dimensions of health, or restricted to health being understood only as the absence of disease. At the next level of conception, health was more multidimensional and there was a continuum from disease to the absence of disease and then to optimum health… The most advanced conception of ‘health that is more than the absence of disease’ was a liberating and expansive way of being there were many positive attributes in all the dimensions of health that were more than just the component parts.”* | Complete wellbeing or functioning, wellbeing, multi-sided |  |
| Johansson et al., 2009 (19) | Sweden | Qualitative research design | Swedish health professionals | *“The analysis of the health professionals' overall views about the concept of health resulted in two categories, "A multi-facetted concept" and "A subjective assessment", which are related to each other. "Health is about life, the whole life" became the third category for the health professional's conceptual framing of health.”* | Multi-sided, subjective, satisfying life | Mental, physical, spiritual |
| Jormfeldt et al., 2009 (20) | Sweden | Cross-sectional study | Patients and staff in mental health services | *“Health is a concept that applies to human beings irrespective of mental disease in the context of mental health services… The most important items according to the staff were ‘‘to experience meaningfulness in life’’ (4.53), ‘‘to experience harmony in life’’ (4.39) ‘‘to be aware of ones worth” (4.36) and ‘‘to feel secure in oneself (4.34)… The item ‘‘to see suffering as a natural part of life’’ was significantly higher rated by the staff than by patients…”* | Satisfying life, self-management |  |
| Lyon, 2012 (21) | USA | Book chapter, conceptual overview | NA | *“Many nurses in practice and nurse educators, however, commonly adopt the biomedical view and equate illness and disease using the terms interchangeably. Likewise, con- cepts of health and wellness are used inter- changeably, logically resulting in the conclusion that persons who have chronic diseases are not and cannot be described as well… Health is defined in many ways within the discipline of nursing (See Table 1.3). Commonly shared attributes of health inherent in all of these definitions, however, is that it is a subjective experience that encompasses how a person is feeling and doing.”* | Complete wellbeing or functioning, subjective |  |
| Merry, 2012 (22) | Canada | Literature study | NA | “… health is viewed from a holistic perspective and involves harmony or balance between body, mind, spirit, and environment (i.e., wholeness). Also, nursing recognizes that each person is unique and that how health is defined by a person, group, or community is subjective.” | Adapting to change, multi-sided, subjective |  |
| Pace et al., 2011 (23) | Italy | Grounded theory approach | Care workers from Italy, South-America, and Eastern Europe | *“All of the participants chose similar labels to define health: they labelled it both as general psycho-physical well-being and as a lack of illness, using phrases such as: “Good health for me is a sense of well-being of ... do not feel any kind of pain, of anxiety” (by the Eastern-European group), or “The health is a good ... I think it is our good mental condition of soul and body, all together” (by the South- American group)… In other words, health seems to be considered as something that is, for the most part, subject to the laws of chance, and of which human beings cannot have full knowledge or complete control.”* | Wellbeing, complete wellbeing or functioning, adapting to change, satisfying life | Mental, physical, individual, environmental |

### Supplementary table 2C: Included articles discussing health from a patient’s perspective

| **Authors, year** | **Country** | **Article type/ study design** | **Perspective (population)** | **Theme Concept of health** | **Subthemes of Concept of health** | **Subthemes of Dimensions of health** |
| --- | --- | --- | --- | --- | --- | --- |
| Bickenbach, 2013 (24) | Switzerland | Literature study | Persons with disabilities | *“But neither the strict social model advocate nor their bête noire, the follower of the medical model, would deny that, whatever else it is, disability is a state or experience of individuals, associated with their bodies and how their bodies function that is often a disadvantage or problem that interferes with their lives and life plans… But the deeper confusion is to assume that a human rights and person’s health state logically depends on their beliefs about what health is.”* | Subjective, daily functioning |  |
| Ebrahimi et al., 2012 (25) | Sweden, USA | Phenomenological approach | Elders in emergency treatment, 80 years and older, or 65 years and older with chronic diseases | *“These frail elders experienced good health when they were able to alter their perceptions, or in other words, shift their fulcrum in such a way as to achieve a harmony and balance of the essential components. The complete experience of health was based on the interactions between two dimensions: the person, and the environment surrounding the person. The threshold of what constituted health was dynamic over time, and depended on the adjustment of the perceived balance point, which was contingent on the elders’ appraisals of their variable life conditions.”* | Subjective, adapting to change | Individual, environmental |
| Gorecki et al., 2010 (26) | United Kingdom | Review of the literature and qualitative approaches | patients with pressure ulcers | No concept of health was written in the article. The article focussed on the dimensions. |  | Physical, mental, functional, social, others |
| Huber et al., 2016 (17) | The Netherlands | Mixed method study, qualitative approach, quantitative approach | Physicians, physiotherapists, policymakers, insurers, public health professionals, researchers, nurses, patients | *“… the new dynamic concept of health as ‘the ability to adapt and to self-manage, in the face of social, physical and emotional challenges’ and elaborated indicators of health … What most respondents generally appreciated about the new concept was that a person is described as more than his illness, and that the focus is on a person’s strength rather than his weakness.”* | Adapting to change, self-management, multi-sided | Functional, physical, mental, social, spiritual, others |
| Hunter et al., 2013 (18) | Australia | Phenomenography method | Patients and practitioners in integrative medicine clinic | *“The simplest conceptions were narrow, limited to physical and mental dimensions of health, or restricted to health being understood only as the absence of disease. At the next level of conception, health was more multidimensional and there was a continuum from disease to the absence of disease and then to optimum health… The most advanced conception of ‘health that is more than the absence of disease’ was a liberating and expansive way of being there were many positive attributes in all the dimensions of health that were more than just the component parts.”* | Complete wellbeing or functioning, wellbeing, multi-sided |  |
| Jormfeldt, 2009 (20) | Sweden | Cross-sectional study | Patients and staff in mental health services | *“The most important items according to the patients were ‘‘to expe- rience harmony in life’’ (4.51), ‘‘to experience meaning- fulness in life’’ (4.51), ‘‘to have a peaceful and positive feeling inside’’ (4.49) and ‘‘to feel hope for the future’’ (4.47)… The item ‘‘to be able to manage ones daily tasks’’ were significantly higher rated by patients than staff, as well as ‘‘being able to trust ones ability’’ and ‘‘to have a peaceful and positive feeling inside’’.”* | Satisfying life, self-management |  |
| Post, 2014 (27) | The Netherlands | Narrative review | NA | *“Whereas health used to be defined primarily in terms of death and the extent of morbidity (ie, disease), the emerging conceptualization of health encompassed how well people function in everyday life and personal evaluations of well- being.”* | Daily functioning, subjective | Physical, mental, social, functional |
| Schrank et al., 2013 (28) | United Kingdom, Austria, Canada | Systematic review and narrative synthesis | People with psychosis | *“Instead the framework places stronger emphasis on individual rather than societal factors, and refers to a number of specific dimensions that may be particularly relevant to people with psychosis, such as mental health and functioning, but also participation, autonomy, self-perception or self-control. In the ONS framework, the domain of individual well-being represents the subjective part of the concept.”* | Daily functioning, participation, self-management, subjective | Individual |
| Shearer et al., 2009 (29) | USA | Qualitative descriptive design | Older women with chronic illness | *“Women defined health consistent with the extent to which they were able to remain active and care for others through their contributions to family and society… Thus, health meant acknowledging this flow of energy, listening to, and respecting its rhythms as part of the meaning and manifestation of health… The women viewed health to a large extent in terms of their ability to make health-related decisions. Health was characterized by a rhythmic pattern of living with the paradox of chronic illness; that is, constructing meanings about one’s health that enhance personal strengths while acknowledging the losses and changes brought on by their illness.”* | Participation, satisfying life, adapting to change, self-management, subjective |  |
| Warsop, 2009 (30) | United Kingdom | Phenomenological approach | NA | *“If we think about health in this, phenomenological, way, we can see that it is not being viewed as a property inhering in human subjects but refers to how we are Being-in-the-world. Health is always in the background, letting us do what we always do. We are always beyond ourselves somehow, involved in the world.”* | Satisfying life, daily functioning |  |
| Zhang et al., 2014 (31) | China | Qualitative descriptive design | Chinese elderly with chronic illness, aged over 60 | *“… ‘Health means not only normal physical function but a responsibility for yourself and others. For my own, it lies in self-maintenance and good quality of life; if lack of quality, long-time living is meaningless.’”* | Multi-sided, self-management |  |

### Supplementary table 2D: Included articles discussing health from the perspective of elderly people

| **Authors, year** | **Country** | **Article type/ study design** | **Perspective (population)** | **Theme Concept of health** | **Subthemes of Concept of health** | **Subthemes of Dimensions of health** |
| --- | --- | --- | --- | --- | --- | --- |
| Boggatz, 2016 (32) | Austria | Concept analysis | Older adults | *“In summary, quality of life in old age in terms of general well-being can be defined as a subjective state characterised by the attributes life satisfaction and emotional balance which mirror the satisfaction of underlying needs and are kept stable by adaptation to worsening life conditions.”* | Subjective, adapting to change, satisfying life |  |
| Cresswell-Smith et al., 2018 (33) | Finland/  Italy/  Norway/ Spain | Rapid review | Older adults, 80 years and older | *“Although there is clear evidence in favour of maintaining physical capability and health in later life, older adults have been seen to adapt and accept limitations as part of the ageing process. Positive thinking and resourcefulness have been identified as mediating challenges, reflected in a tendency towards defining health in relation to abilities, not in terms of symptoms and disease. These findings underline the need to support functional ability despite the existence of ill health, encouraging participation, and defining wellbeing via functionality and ability rather than on impairments.”* | Adapting to change, self-management, daily functioning | Functional, social, individual, environmental |
| Ebrahimi et al., 2012 (25) | Sweden, USA | Phenomenological approach | Elders in emergency treatment, 80 years and older, or 65 years and older with chronic diseases | *“These frail elders experienced good health when they were able to alter their perceptions, or in other words, shift their fulcrum in such a way as to achieve a harmony and balance of the essential components. The complete experience of health was based on the interactions between two dimensions: the person, and the environment surrounding the person. The threshold of what constituted health was dynamic over time, and depended on the adjustment of the perceived balance point, which was contingent on the elders’ appraisals of their variable life conditions.”* | Subjective, adapting to change | Individual, environmental |
| Fange & Ivanoff, 2009 (34) | Sweden | Grounded theory method | Old age, between 80-89 years old | *“Health was described in terms of being able to manage daily activities at home and to participate in society… Health was very much related to the possibility of being active and participating in social life in keeping with one’s own preferences, and it was always evaluated in relation to their age and what they perceived could be expected in this context.”* | Participation, self-management |  |
| Goins et al., 2011 (35) | USA | Qualitative approach | community dwelling persons aged 60 years or older in west Virginia | *“… defining health as a value indicates it can be fleeting, both lost and regained… According to the participants, health cannot be compartmentalized but includes elements of physical, behavioral, psychological, and spiritual well-being… Our results expand on previous studies and demonstrate that health is a subjective, multidimensional construct deeply embedded in the everyday experience of Appalachian rural elders… Health can be conceptualized as the capacity to perform certain tasks and fulfill societal roles. Practicing healthy behaviors is one way that rural older adults can maintain good health and continue to function independently.”* | Participation, subjective, adapting to change, satisfying life, multi-sided | Physical, functional, mental, spiritual |
| Noghabi et al., 2013 (36) | Iran | Theoretical analysis of literature and empirical observation. Hybrid concept analysis. | Old people, 65 years and older | *“Health among the old is a concept that is affected by genetic, environmental, healthcare services and lifestyle-related factors and involves proportional physical, mental, social, familial, spiritual, and economical welfare along with the ability to handle daily life activities which is measurable through medical and functional approaches.”* | Self-management | Physical, mental, social, spiritual, environmental |
| Shearer et al., 2009 (29) | USA | Qualitative descriptive design | Older women with chronic illness | *“Women defined health consistent with the extent to which they were able to remain active and care for others through their contributions to family and society… Thus, health meant acknowledging this flow of energy, listening to, and respecting its rhythms as part of the meaning and manifestation of health… The women viewed health to a large extent in terms of their ability to make health-related decisions. Health was characterized by a rhythmic pattern of living with the paradox of chronic illness; that is, constructing meanings about one’s health that enhance personal strengths while acknowledging the losses and changes brought on by their illness.”* | Participation, satisfying life, adapting to change, self-management, subjective |  |
| Song & Kong, 2015 (37) | Republic of Korea | Systematic review | Older adults | *“This meta-study found that older adults experience health when they have the ability to do something independently, absence or management of symptoms, acceptance and adjustment with optimism, connectedness with others, and enough energy in their own world.”* | Self-management, adapting to change, satisfying life | Physical, mental, social, spiritual |
| Zhang et al., 2014 (31) | China | Qualitative descriptive design | Chinese elderly with chronic illness | *“… ‘Health means not only normal physical function but a responsibility for yourself and others. For my own, it lies in self-maintenance and good quality of life; if lack of quality, long-time living is meaningless.’”* | Multi-sided, self-management |  |

### Supplementary table 2E: Included articles discussing health from a philosophical perspective

| **Authors, year** | **Country** | **Article type/ study design** | **Perspective (theoretical approach)** | **Theme Concept of health** | **Subthemes of Concept of health** | **Subthemes of Dimensions of health** |
| --- | --- | --- | --- | --- | --- | --- |
| Included articles discussing health from a social science perspective | | | | | | |
| Bauer et al., 2020 (38) | Switzerland, Canada, Kenya, Italy, United Kingdom, Sweden, Norway, Denmark, Spain, Israel, Austria, Singapore, Netherlands, | Literature study | Salutogenic | Discussing feature developments for the salutogenic model of health. |  |  |
| Bircher & Kuruvilla, 2014 (39) | Switzerland | Multi-grounded theory method | Multi-grounded theory | *“Health is a state of wellbeing emergent from conducive interactions between individuals’ potentials, life’s demands, and social and environmental determinants… The Meikirch Model of Health represents health as a complex adaptive system containing ongoing interactions between individuals’ potentials, the demands of life, and social and environmental determinants.”* | Wellbeing, adapting to change, multi-sided | Environmental, individual, social |
| Cloninger et al., 2012 (40) | USA | Literature study | Holistic | *“… health is better conceptualized as a complex adaptive system that is dynamically self-organizing within a person as s/he adapts to an ever-changing internal and external environment, rather than a collection of independent diseases.”* | Multi-sided, adapting to change |  |
| de Araújo et al. 2012 (41) | Brazil | Theoretical study | Hermeneutics | *“Health is presented as a state of balance and, as such, something based on the experience of the ‘being’ in the face of the world of life, which is his/her everyday life, constructed from which elements are brought by culture, inter-subjectivity and language.”* | Subjective, adapting to change |  |
| Elliot, 2016 (42) | United Kingdom | Literature study | Eudaimonistic | *“… health is not just something to be located in organs, vital signs, and bodily functions, but take a more comprehensive view that includes mental and psychological health.”* | Multi-sided |  |
| Ereshefsky, 2009 (43) | Canada | Paper | Naturalist/ normativist | Naturalist, normativist and hybrid theories of health were explored. |  | Physical, mental |
| Haverkamp et al., 2018 (44) | The Netherlands | Practice-oriented review | Philosophical | Five different concepts of health were compared. Health as the ability to function normally and biologically (Boorse). Health as the (second-order) ability to achieve vital goals (Nordenfelt). Health as the meta-capability to achieve a set of basic capabilities (Venkatapuram). Health as overall physical, mental, and social wellbeing (WHO). Health as the ability to adapt and self-manage (Huber et al.) |  |  |
| Huber et al. 2011 (45) | The Netherlands | Analysis | Positive health | *“The first step towards using the concept of “health, as the ability to adapt and to self-manage” is to identify and characterise it for the three domains of health: physical, mental, and social.”* | Adapting to change, self-management | Physical, mental, social |
| Leonardi, 2018 (46) | Italy | Literature study | Epistemological | *“This newly proposed definition configures health as the capability to cope with and to manage one’s own malaise and well-being conditions. In more operative terms, health may be conceptualized as the capability to react to all kinds of environmental events having the desired emotional, cognitive, and behavioral responses and avoiding those undesirable ones.”* | Self-management, adapting to change, daily functioning |  |
| Misselbrook, 2014 (47) | Bahrain | Note | Human flourishing | *“Thus health can be seen as the ability to flourish without being unduly impeded by illness or disability or, if necessary, by overcoming illness or disability… Health care should aim for the state of least possible illness or disability, or of maximal functional adaptation to illness or disability.”* | Satisfying life, adapting to change |  |
| Misselbrook, 2016 (48) | Bahrain | Literature study | Human flourishing | *“Thus, health can be seen as the ability to flourish without being unduly impeded by illness or disability or, if necessary, by overcoming illness or disability… But if we truly believe in a multidimensional model of health, which includes the biomedical, social, psychological, anthropological and spiritual dimensions, then we are swimming against the stream.”* | Satisfying life, multi-sided, adapting to change | Physical, mental, social, spiritual, others |
| Prinsen & Terwee, 2019 (49) | The Netherlands | Mixed-method study including a literature search, a qualitative and quantitative ranking study, followed by a content validity study | Positive health | Huber’s positive health concept was criticised. |  |  |
| Reed, 2019 (50) | USA | Review | Philosophical | *“These theoretical foundations in the philosophy of health, classic and contemporary, point to an expanded conceptualization of health that integrates two key dimensions: naturalism (where health is regarded as something real about which there are facts that are meaningful in everyday life, and including psychosocial and other health processes as well as the typically biological or physical) and normative (values and norms of both the individual and society).”* | Subjective, satisfying life | Physical, social |
| Van Spijk, 2015 (51) | Switzerland | Scientific contribution | Philosophical anthropology | *“Human health—also called ‘great health’—is the ability to live a life that makes sense… ‘Great health’ refers to the field that opens up whenever a person is able to live life to the full and therefore regularly comes up against existential boundaries. By doing so, the feeling of sense may arise.”* | Satisfying life |  |
| Sturmberg et al., 2010 (52) | Australia/USA | Literature study | Philosophical | *“The perception of being healthy is an emergent phenomenon based on individual and collective understandings of everyday realities. Perceiving health in this way requires embracing a complex adaptive systems approach, rather than the “rational” approach of the ruling biomedical model… Health is a dynamic state: it is neither solely an individual construction (illness), a reflection of societal attributes (sickness), nor a body or mind clinicopathologic system in varying degrees of order and disorder (disease).”* | Subjective, adapting to change, multi-sided | Physical, mental, social, functional |
| Sturmberg, 2014 (53) | Australia | Commentary | Philosophical | *“The basic proposition that health is the emergent product of a dynamic nonlinear interaction between the "biological potentials", "acquired potentials", and the "demands of life" is a useful model to conceptualise the nature of health.”* | Adapting to change |  |
| Tengland, 2016 (54) | Sweden | Critical discussion | Holistic/ capability approach | *“… every theory of health has to take subjective wellbeing into account, in one way or another, either as a causal factor, as Nordenfelt does, or as conceptually related to health, as I have argued. This, however, is an aspect of health about which Venkatapuram remains silent… I endorse a holistic theory as a general approach for defining health.”* | Subjective, wellbeing, multi-sided | Environmental |
| Tyreman, 2011 (55) | United Kingdom | Literature study | Phenomenological/ hermeneutics | *“The recognition that health is more than the absence and illness more than the symptoms of disease… On this interpretation, health is not a state or process, but a relationship premised on how we connect with the world and entailing how we perceive the world from one direction and experience it in return. In other words health is the holistic and ontological phenomenon of engagement… health is not just about well-being, it is also about having resources to adapt to situations one finds oneself in… Health is the retentir that emanates from being, from our dynamic participation in the World. It is a unique, ever-changing phenomenological ontology that gives meaning to our world and is the ground of Dasein.”* | Multi-sided, subjective, adapting to change, participation |  |
| Venkatapuram, 2013 (56) | United Kingdom | Debate | Capability approach | *“… the concept of health for many people does not only relate to the presence or absence of disease. It has to do with how they feel and what they are able to do… health as a concept can be defensibly conceived as a meta-capability, the capability to achieve a cluster of basic capabilities to be and do things that reflect a life worthy of equal human dignity.”* | Daily functioning, subjective, satisfying life |  |
| Included articles discussing health from a biomedical science perspective | | | | | | |
| Boorse, 2011 (57) | USA | Conceptual analysis | Naturalist | *“Health in a member of the reference class is normal functional ability: the readiness of each internal part to perform all its normal functions on typical occasions with at least typical efficiency.”* | Complete wellbeing or functioning |  |
| Boorse, 2014 (58) | USA | Reactions to critics | Naturalist | *“… the BST, which defines health as normal physiological functional ability… The BST requires all parts of a healthy organism, including cells and organelles, to have species-typical functional readiness.”* | Complete wellbeing or functioning |  |
| Hafen, 2016 (59) | Switzerland | Sociological systems theory | Constructivist | *“… it is strongly recommended to define health merely as the absence of disease or infirmity and put the distinction of health/health impairments carefully in relation to other distinctions that describe the conditions and consequences of health without integrating them in the term 'health'.”* | Complete wellbeing or functioning |  |
| Schroeder, 2013 (60) | United Kingdom | Literature study | Comparative | *“A large majority of the theories of health on offer could be described as broadly functionalist in the following respect: they declare an organism healthy based on whether the organism (or some part of the organism) can do something.”* | Daily functioning |  |

### Supplementary table 2F: Included articles discussing health from a theological perspective

| **Authors, year** | **Country** | **Article type/ study design** | **Perspective (theoretical approach or population)** | **Theme Concept of health** | **Subthemes of Concept of health** | **Subthemes of Dimensions of health** |
| --- | --- | --- | --- | --- | --- | --- |
| Messer, 2013 (61) | United Kingdom | Philosophical discussion, book chapter | Theological | *“A theological understanding of human beings as the creatures of a good and loving God is likely to prove receptive to a way of conceptualizing health that locates it within an understanding of the goods, goals, and ends of human creaturely life… If human creaturely flourishing is understood in theological terms, then it seems promising to conceptualize health as the capacity to realize (some aspects of) that creaturely flourishing.”* | Satisfying life |  |
| Proeschold-Bell et al., 2009 (62) | USA | Grounded theory approach | United Methodist church pastors | *“In this model, we define our final health outcome holistically to indicate that health is not merely the absence of problems but is, rather, the presence of multiple life satisfactions. The outcome thus includes not only physical and mental health and spiritual well-being, but also the overall quality of life… The participants defined health as, ‘‘wholeness of the spirit. Mind, body, and spirit’’; ‘‘a general sense of well-being’’; and ‘‘spiritual, emotional, physical, mental well-being.’’”* | Multi-sided, satisfying life, wellbeing | Physical, mental, spiritual, others |
| Sadat Hoseini et al., 2015 (63) | Iran | Concept analysis | Islamic philosophy | *“However, according to the Islamic definition, the concept of health is defined based on the process that individuals go through during illness and health… It seems that the concept of health in Islam is very similar to nursing theories and through a holistic view, they all consider that the concept of health is dependent upon human consciousness and wisdom, and accordingly, emphasize superiority of spiritual health over physical health.”* | Adapting to change, multi-sided | Physical, mental, social, spiritual |
| Tirodkar et al., 2010 (64) | USA | Qualitative research design | South Asian immigrants in Chicago / religion | *“In this study, we found that South Asian immigrants’ EMs for health and disease were largely conceptualized within a bio-psychosocial framework. However, it is notable that one third of the participants (the majority of whom were Muslim participants) endorsed a holistic model of health that also encompassed spiritual factors.”* | Multi-sided | Functional, social, physical, spiritual |
| Walther et al., 2015 (65) | Kenya/USA | Phenomenological approach | United Methodist Church clergy | *“The clergy defined health holistically, not merely an absence of disease or physical illness, but rather including physical health and spiritual and emotional well-being.”* | Multi-sided, wellbeing | Physical, mental, spiritual, environmental |

### Supplementary table 2G: Included articles discussing health from a context specific perspective

| **Authors, year** | **Country** | **Article type/ study design** | **Perspective (population)** |  | **Subthemes of Concept of health** | **Subthemes of Dimensions of health** |
| --- | --- | --- | --- | --- | --- | --- |
| Included articles discussing health from a cultural specific perspective | | | | | | |
| Kendall et al., 2019 (66) | Australia | Community collaborative participatory action research | Aboriginal mothers in metropolitan regional, and remote prisons | *“Women said that health was about the absence of health problems, overcoming health problems, and getting off or maintaining desistance from harmful substances… Women conceptualized health as a process and highlighted the role of agency in a “becoming healthy process”. Becoming healthy was a sequential process with discrete steps that needed to be negotiated over time. Women recognized the need for action and repetition of action in the becoming healthy process… The women in this study offered an alternative to individualized, diseasemodel approaches to health, describing a holistic concept of health as inextricable from their culture, identity, histories, and social and structural circumstances intertwined with those of their family and community.”* | Complete wellbeing or functioning, adapting to change, self-management, multi-sided |  |
| Mark & Lyons., 2010 (67) | New Zealand | Phenomenological approach | Māori spiritual healers | *“In summary, this study revealed principles of Mäori healing that originate from ancient cultural knowledge and traditions which see the mind, body, spirit, family, and land as essential aspects of health and wellbeing. The connectedness of mind, body, and spirit was highlighted, but the external relationships people have with their family/genealogy and with the land are viewed as just as important for maintaining good health.”* | Multi-sided, satisfying life | Spiritual, environmental, others |
| Seyedfatemi et al., 2014 (68) | Iran | Systematic review | Iranian women’s health concepts | *“The present study showed that women’s health personal factors include dimensions such as physical, psycho- social and spiritual. It also showed that health is only feasible when balance is among these dimensions.”* | Multi-sided, adapting to change | Environmental, social, individual: physical, social, spiritual |
| Yang et al., 2016 (69) | Republic of Korea/USA | Qualitative method | Nepalese women, had lived in the Dadeldhura district for more than 5 years | *“Our findings suggest that the women’s concept of health can be summarized into four categories of meaning: absence of disease, no tension, peace in the family, and being able to work.”* | Complete wellbeing or functioning, satisfying life, participation |  |
| Included articles discussing health from an immigrant’s perspective | | | | | | |
| Cha, 2013 (70) | South-Korea | Grounded theory method | Korean migrant women who migrated to North-America or Canada for their children’s education while their husbands remained in Korea | *“Being healthy meant being able to take care of their children; therefore, health became a prerequisite to a goose mother’s survival in a foreign country. Surveys examining the goose mothers’ health conception corresponded to this newly developed concept. Goose mothers agreed the most with functional health… Their definition of health is limited to not getting sick, and continuing to be able to take care of their children.”* | Satisfying life, daily functioning, complete wellbeing or functioning |  |
| Martin, 2009 (71) | USA | Phenomenology | Older Iranian immigrants | *“Many of the participants described ‘‘being healthy’’ as a state of having balance in their lives; that is, they had the proper combination of mental/emotional, physical, and spiritual health as well as a balanced diet and social life. In general, they defined health as an overall absence of worry, good nutrition, ‘‘happy hearts,’’ and a feeling of being connected to their family and community… Iranian immigrants define health holistically (including the mind, body, emotions, and spirit), and, hence, they expect their health providers to care for them as a whole person and to not just focus on illness/disease elimination.”* | Adapting to change, multi-sided | Mental, physical, spiritual, social, others |
| Tirodkar et al., 2011 (64) | USA | Qualitative research design | South Asian immigrants in Chicago / religion | *“In this study, we found that South Asian immigrants’ EMs for health and disease were largely conceptualized within a bio-psychosocial framework. However, it is notable that one third of the participants (the majority of whom were Muslim participants) endorsed a holistic model of health that also encompassed spiritual factors.”* | Multi-sided | Functional, social, physical, spiritual |
| Included articles discussing health from an educational perspective | | | | | | |
| Jensen, 2013 (72) | Denmark | Qualitative approach | Women with low levels of education | *“On a general level, the women share a particular understanding of what health is. When answering the question ‘what is health?’ they reproduce the prevalent official health advice regarding diet, smoking, alcohol and exercise. Nevertheless, by probing deeper into their accounts, it becomes clear that this bio-medical interpretation of health is more or less entirely removed from their lives and reference frames... The accounts above suggest that the women share an understanding of health being closely related to ‘the good life’ and a sense of well-being… Evidently, the interview accounts demonstrate that health, for these women, is tied to the quality of life and being able to live the good life.”* | Wellbeing, complete wellbeing or functioning, multi-sided, satisfying life |  |
| Stronks et al., 2018 (73) | The Netherlands | Concept mapping | Lay persons with a lower educational le*v*el | *“In all three groups health was conceptualised as a multidimensional concept. Four clusters occurred in all groups, that is, absence of disease and functioning, health-related behaviours, social life and attitude towards life.” Differences were also observed. First, some dimensions appeared to be specific for particular educational groups, for example, access to good healthcare was specific for the lower/intermediate education group, autonomy or independence for the intermediate/higher education group and perceived health for the higher education group. Second, the content of some clusters differed. The cluster ‘absence of disease’ was literally used in the lower education group, but was broadened to ‘functioning’ in the intermediate education group, and further broadened to ‘self-perceived health’ in the higher education group. Social life was conceptualised in the lower education group in terms of ‘having a nice time together’ to ‘having meaningful relationships’ in the higher education group. ‘Attitude towards life’ in the higher educational group suggests ‘lust for life’, and shifted to an emphasis on satisfaction and acceptance of life in the lower education group.”* | Complete wellbeing or functioning, daily functioning, multi-sided, satisfying life |  |
|  |  |  | Lay persons with an intermediate educational level |  | Complete wellbeing or functioning, daily functioning, multi-sided, satisfying life, self-management, |  |
|  |  |  | Lay persons with an higher educational level |  | Complete wellbeing or functioning, daily functioning, multi-sided, satisfying life, subjective, self-management |  |
| Included articles discussing health from other context specific perspectives | | | | | | |
| Mayer & Bones, 2011 (74) | Germany, South-Africa | Multi-method research | South-African managers and expatriates | *“Most of the managers refer to health as a positive concept of well-being, and only a few managers view health in a ‘‘pathogenic’’ way as ‘‘absence of sickness’’. They rather refer to a ‘‘salutogenic’’ health concept that focuses on their personal and social resources. Health is interlinked with individual (life) energy and happiness rather than with work performance.”* | Wellbeing, multi-sided, subjective | Mental, physical, spiritual |
| Rawolle et al., 2016 (75) | Australia | Descriptive qualitative study | South-Australian farmers | *Farmers perceive health as being able to function and work on the farm… At the individual level, participants described health as the capacity to function and participate fully in daily life… Many participants considered health as a condition to be fixed as the need arose, to restore functional capacity. Health from a functional perspective was seen as analogous to farm machinery.”* | Daily functioning, participation, complete wellbeing or functioning | Individual, social, environmental |

## References

1. Abuelaish I, Goodstadt MS, Mouhaffel R. Interdependence between health and peace: a call for a new paradigm. Health Promot Int. 2020;1–11.

2. Amzat J, Razum O. Health, Disease, and Illness as Conceptual Tools. In: Medical sociology in Africa. 2014. p. 21–37.

3. Conner AL, Boles DZ, Markus HR, Eberhardt JL, Crum AJ. Americans’ Health Mindsets: Content, Cultural Patterning, and Associations With Physical and Mental Health. Ann Behav Med. 2019;53(4):321–32.

4. Downey CA, Chang EC. Assessment of everyday beliefs about health: The Lay Concepts of Health Inventory, college student version. Psychol Health. 2013;28(7):818–32.

5. Frenk J, Gómez-Dantés O. Designing a framework for the concept of health. J Public Health Policy. 2014;35(3):401–6.

6. Kaldjian LC. Concepts of health, ethics, and communication in shared decision making. Commun Med. 2017;14(1):83–95.

7. Karimi M, Brazier J. Health, Health-Related Quality of Life, and Quality of Life: What is the Difference? Pharmacoeconomics. 2016;34(7):645–9.

8. Lipworth WL, Hooker C, Carter SM. Balance, Balancing, and Health. Qual Health Res. 2011;21(5):714–25.

9. Makoul G, Clayman ML, Lynch EB, Thompson JA. Four Concepts of Health in America: Results of National Surveys. J Health Commun. 2009;14(1):3–14.

10. Pietersma S, de Vries M, van den Akker-van Marle ME. Domains of quality of life: results of a three-stage Delphi consensus procedure among patients, family of patients, clinicians, scientists and the general public. Qual Life Res. 2013;

11. Shilton T, Sparks M, McQueen D, Lamarre MC, Jackson S. Proposal for new definition of health. Bmj. 2011;343(aug23 4):d5359–d5359.

12. Thumboo J, Ow MYL, Uy EJB, Xin X, Chan ZYC, Sung SC, et al. Developing a comprehensive, culturally sensitive conceptual framework of health domains in Singapore. PLoS One. 2018;13(6):e0199881.

13. Williamson DL, Carr J. Health as a resource for everyday life: advancing the conceptualization. Crit Public Health. 2009;19(1):107–22.

14. Alslman ET, Ahmad MM, Bani Hani MA, Atiyeh HM. Health: A Developing Concept in Nursing. Int J Nurs Knowl. 2017;28(2):64–9.

15. Ashcroft R, Van Katwyk T. Joining the Global Conversation: Social Workers Define Health Using a Participatory Action Research Approach. Br J Soc Work. 2016;bcw005.

16. Bąk-Sosnowska M, Skrzypulec-Plinta V. Health behaviors, health definitions, sense of coherence, and general practitioners’ attitudes towards obesity and diagnosing obesity in patients. Arch Med Sci. 2017;2:433–40.

17. Huber M, van Vliet M, Giezenberg M, Winkens B, Heerkens Y, Dagnelie PC, et al. Towards a patient-centred operationalisation of the new dynamic concept of health. Br Med J open. 2016;6(1):1–12.

18. Hunter J, Marshall J, Corcoran K, Leeder S, Phelps K. A positive concept of health – Interviews with patients and practitioners in an integrative medicine clinic. Complement Ther Clin Pract. 2013;19(4):197–203.

19. Johansson H, Weinehall L, Emmelin M. ‘It depends on what you mean’: a qualitative study of Swedish health professionals’ views on health and health promotion. BMC Health Serv Res. 2009;9(1).

20. Jormfeldt H. Attitudes towards health among patients and staff in mental health services: a comparison of ratings of importance of different items of health. Soc Psychiatry Psychiatr Epidemiol. 2009;45(2):225–31.

21. Lyon BL. Stress, Coping, and Health, a conceptual overview. In: Handbook of stress, coping, and health: Implications for nursing research, theory, and practice. Sage Publications, Inc; 2012. p. 2–20.

22. Merry L. Global health for nursing...and nursing for global health. Can J Nurs Res. 2012;44(4):20–35.

23. Pace CS, Velotti P, Zavattini GC. Representations of health and illness by Eastern European, South American and Italian care workers: a qualitative study. J Health Psychol. 2011;17(4):490–9.

24. Bickenbach J. Being unhealthy, and rights to health. J law, Med ethics. 2013;41(4):821–8.

25. Ebrahimi Z, Wilhelmson K, Moore CD, Jakobsson A. Frail Elders’ Experiences With and Perceptions of Health. Qual Health Res. 2012;22(11):1513–23.

26. Gorecki C, Lamping DL, Brown JM, Madill A, Firth J, Nixon J. Development of a conceptual framework of health-related quality of life in pressure ulcers: A patient-focused approach. Int J Nurs Stud. 2010;47(12):1525–34.

27. Post M. Definitions of Quality of Life: What Has Happened and How to Move On. Top Spinal Cord Inj Rehabil. 2014;20(3):167–80.

28. Schrank B, Bird V, Tylee A, Coggins T, Rashid T, Slade M. Conceptualising and measuring the well-being of people with psychosis: Systematic review and narrative synthesis. Soc Sci Med. 2013;92:9–21.

29. Shearer NBC, Fleury JD, Reed PG. The Rhythm of Health in Older Women With Chronic Illness. Res Theory Nurs Pract. 2009;23(2):148–60.

30. Warsop A. Medically unexplained symptoms and the meaning of health, a phenomenological clue. Psychiatry. 2009;8(5):149–52.

31. Zhang H, Shan W, Jiang A. The meaning of life and health experience for the Chinese elderly with chronic illness: A qualitative study from positive health philosophy. Int J Nurs Pract. 2014;20(5):530–9.

32. Boggatz T. Quality of life in old age - a concept analysis. Int J Older People Nurs. 2016;11(1):55–69.

33. Cresswell-Smith J, Amaddeo F, Donisi V, Forsman AK, Kalseth J, Martin-Maria N, et al. Determinants of multidimensional mental wellbeing in the oldest old: a rapid review. Soc Psychiatry Psychiatr Epidemiol. 2018;54(2):135–44.

34. Fänge A, Ivanoff SD. The home is the hub of health in very old age: Findings from the ENABLE-AGE Project. Arch Gerontol Geriatr. 2009;48(3):340–5.

35. Goins RT, Spencer SM, Williams K. Lay Meanings of Health Among Rural Older Adults in Appalachia. J Rural Heal. 2011;27(1):13–20.

36. Noghabi AA, Alhani F, Peyrovi H. Health Hybrid Concept Analysis in Old People. Glob J Health Sci. 2013;5(6).

37. Song M, Kong E-H. Older adults’ definitions of health: A metasynthesis. Int J Nurs Stud. 2015;52(6):1097–106.

38. Bauer GF, Roy M, Bakibinga P, Contu P, Downe S, Eriksson M, et al. Future directions for the concept of salutogenesis: a position article. Health Promot Int. 2020;35(2):187–95.

39. Bircher J, Kuruvilla S. Defining health by addressing individual, social, and environmental determinants: New opportunities for health care and public health. J Public Health Policy. 2014;35(3):363–86.

40. Cloninger R, Salloum IM, Mezzich JE. The dynamic origins of positive health and wellbeing. Int J Pers Cent Med. 2012;2(2):179–87.

41. de Araújo JL, Araujo Paz EP, Moreira TMM. Hermeneutics and health, reflections on the thinking of Hans-Georg Gadamer. Rev da Esc Enferm da USP. 2012;46(1):194–201.

42. Elliot D. Defining the Relationship Between Health and Well-being in Bioethics. New Bioeth. 2016;22(1):4–17.

43. Ereshefsky M. Defining ‘health’ and ‘disease’. Stud Hist Philos Sci Part C Stud Hist Philos Biol Biomed Sci. 2009;40(3):221–7.

44. Haverkamp B, Bovenkerk B, Verweij MF. A Practice-Oriented Review of Health Concepts. J Med Philos A Forum Bioeth Philos Med. 2018;43(4):381–401.

45. Huber M, Knottnerus JA, Green L, Horst H v d, Jadad AR, Kromhout D, et al. How should we define health? Bmj. 2011;343(jul26 2):d4163–d4163.

46. Leonardi F. The Definition of Health: Towards New Perspectives. Int J Heal Serv. 2018;48(4):735–48.

47. Misselbrook D. W is for Wellbeing and the WHO definition of health. Br J Gen Pract. 2014;64(628):582.

48. Misselbrook D. Aristotle, Hume and the goals of medicine. J Eval Clin Pract. 2016;22(4):544–9.

49. Prinsen CAC, Terwee CB. Measuring positive health: for now, a bridge too far. Public Health. 2019;170:70–7.

50. Reed PG. Adaptive Preferences: A Philosophical Issue Raised by an Expanded Model of Health. Nurs Sci Q. 2019;32(3):201–6.

51. van Spijk P. On human health. Med Heal Care Philos. 2014;18(2):245–51.

52. Sturmberg JP, Martin CM, Moes MM. Health at the Center of Health Systems Reform: How Philosophy Can Inform Policy. Perspect Biol Med. 2010;53(3):341–56.

53. Sturmberg JP. Emergent properties define the subjective nature of health and disease. J Public Health Policy. 2014;35(3):414–9.

54. Tengland P-A. Venkatapuram’s Capability theory of Health: A Critical Discussion. Bioethics. 2016;30(1):8–18.

55. Tyreman S. The happy genius of my household: phenomenological and poetic journeys into health and illness. Med Heal Care Philos. 2011;14(3):301–11.

56. Venkatapuram S. Health, Vital Goals, and Central Human Capabilities. Bioethics. 2013;27(5):271–9.

57. Boorse C. Concepts of Health and Disease. 2011;13–64.

58. Boorse C. A Second Rebuttal On Health. J Med Philos. 2014;39(6):683–724.

59. Hafen M. Of what use (or harm) is a positive health definition? J Public Health (Bangkok). 2016;24(5):437–41.

60. Schroeder SA. Rethinking Health: Healthy or Healthier than? Br J Philos Sci. 2012;64(1):131–59.

61. Messer N. Philosophical Accounts of Health, Disease, and Illness. In: Flourishing : Health, disease, and bioethics in theological perspective. William B. Eerdmans publishing company; 2013. p. 1–50.

62. Proeschold-Bell RJ, LeGrand S, James J, Wallace A, Adams C, Toole D. A Theoretical Model of the Holistic Health of United Methodist Clergy. J Relig Health. 2009;50(3):700–20.

63. Sadat Hoseini AS, Khosro Panah AH, Alhani F. The Concept Analysis of Health Based on islamic sources, intellectual health. Int J Nurs Knowl. 2015;26(3):113–20.

64. Tirodkar MA, Baker DW, Makoul GT, Khurana N, Paracha MW, Kandula NR. Explanatory Models of Health and Disease Among South Asian Immigrants in Chicago. J Immigr Minor Heal. 2010;13(2):385–94.

65. Walther NG, Proeschold-Bell RJ, Benjamin-Neelon S, Adipo S, Kamaara E. “We Hide Under the Scriptures”: Conceptualization of Health Among United Methodist Church Clergy in Kenya. J Relig Health. 2014;54(6):2235–48.

66. Kendall S, Lighton S, Sherwood J, Baldry E, Sullivan E. Holistic Conceptualizations of Health by Incarcerated Aboriginal Women in New South Wales, Australia. Qual Health Res. 2019;29(11):1549–65.

67. Mark GT, Lyons AC. Maori healers’ views on wellbeing: The importance of mind, body, spirit, family and land. Soc Sci Med. 2010;70(11):1756–64.

68. Seyedfatemi N, Salsali M, Rezaee N, Rahnavard Z. Women’s Health Concept, A Meta-Synthesis Study. Iran J Public Health. 2014;43(10):1335–44.

69. Yang Y, Bekemeier B, Choi J. A cultural and contextual analysis of health concepts and needs of women in a rural district of Nepal. Glob Health Promot. 2016;25(1):15–22.

70. Cha C. Health Concept and Health Promotion Process Among Korean Migrant Women. Health Care Women Int. 2013;34(8):628–50.

71. Martin SS. Healthcare-Seeking Behaviors of Older Iranian Immigrants: Health Perceptions and Definitions. J Evid Based Soc Work. 2009;6(1):58–78.

72. Jensen JM. Everyday life and health concepts among blue-collar female workers in Denmark: implications for health promotion aiming at reducing health inequalities. Glob Health Promot. 2013;20(2):13–21.

73. Stronks K, Hoeymans N, Haverkamp B, den Hertog FRJ, van Bon-Martens MJH, Galenkamp H, et al. Do conceptualisations of health differ across social strata? A concept mapping study among lay people. BMJ Open. 2018;8(4):e020210.

74. Mayer C-H, Boness C. Concepts of health and well-being in managers: An organizational study. Int J Qual Stud Health Well-being. 2011;6(4):7143.

75. Rawolle TA, Sadauskas D, van Kessel G, Dollman J. Farmers’ perceptions of health in the Riverland region of South Australia: ‘If it’s broke, fix it’’’. Aust J Rural Health. 2016;24(5):312–6.
