# Supplementary material for: Concepts of health in different contexts: a scoping review
Source: BMC Health Serv Res. 2022 Mar 24;22:389. doi: 10.1186/s12913-022-07702-2 (PMC8953139; doi:10.1186/s12913-022-07702-2)
Supplement: Supplementary file 3 — Additional file 3: Supplementary Table 3A. Overview of number of articles per subtheme for theme 1 (concept of health) for different perspectives. Supplementary Table 3B. Overview of number of articles per subtheme for theme 2 (dimensions of health) for different perspectives. [file 12913_2022_7702_MOESM3_ESM.docx]

**Supplementary table 3A: overview of number of articles per subtheme for theme 1 (concept of health) for different perspectives.**

| **Subtheme of Perspective (theme 3) 🡪** | **General** | **Care workers** | **Patients** | **Older people** | **Philosophical (social)** | **Philosophical (biomedical)** | **Theological** |
| --- | --- | --- | --- | --- | --- | --- | --- |
| **Number of articles** | 13 | 10 | 11 | 9 | 19 | 4 | 5 |
| **Subtheme of concept of health (theme 1)**  **🡫** | | | | | | | |
| **Multi-sided**  (Health is not related only to the physical dimension, but involves several dimensions) | 5 | 6 | 3 | - | 7 | - | 4 |
| **Self-management**  (Having self-control in life and in the health process) | 3 | 2 | 4 | 6 | 2 | - | - |
| **Participation**  (Being active and participating in life) | 3 | - | 2 | 4 | 1 | - | - |
| **Adapting to change**  (Being able to adapt to personal or environmental health-related changes and circumstances) | 2 | 3 | 3 | 6 | 10 | - | 1 |
| **Subjective**  (Personal perceptions and experiences about health) | 3 | 4 | 5 | 4 | 5 | - | - |
| **Satisfying life**  (Values that contribute to satisfaction in life) | 2 | 3 | 3 | 4 | 5 | - | 2 |
| **Wellbeing**  (Wellbeing in several ways but not referring to complete wellbeing or functioning) | - | 3 | 1 | - | 2 | - | 2 |
| **Daily functioning**  (Functioning in life) | 2 | - | 4 | 1 | 2 | 1 | - |
| **Complete wellbeing or functioning**  (Functioning without any disturbance of diseases or infirmities, for example ‘absence of disease’) | 2 | 3 | 1 | - | - | 3 | - |

^For example, there were ten articles discussing the concept of health from the care workers perspective. The subtheme multi-sided was represented in six of these articles.^

**Supplementary table 3B: overview of number of articles per subtheme for theme 2 (dimensions of health) for different perspectives.**

| **Subtheme of Perspective (theme 3) 🡪** | **General** | **Care workers** | **Patients** | **Older people** | **Philosophical (social)** | **Philosophical (biomedical)** | **Theological** |
| --- | --- | --- | --- | --- | --- | --- | --- |
| **Number of articles** | 13 | 10 | 11 | 9 | 19 | 4 | 5 |
| **Subtheme of dimensions of health (theme 1)**  **🡫** | | | | | | | |
| **Physical** | 5 | 5 | 3 | 3 | 5 | - | 4 |
| **Mental** | 5 | 5 | 3 | 3 | 4 | - | 3 |
| **Social** | 6 | 3 | 3 | 3 | 5 | - | 2 |
| **Spiritual** | 4 | 3 | 1 | 3 | 1 | - | 4 |
| **Functional** | 2 | 1 | 3 | 2 | 1 | - | 1 |
| **Environmental** | 2 | 2 | 1 | 3 | 2 | - | 1 |
| **Individual** | - | 1 | 2 | 2 | 1 | - | - |
| **Others** | 2 | 1 | 1 | - | 1 | - | 1 |

^For example, there were ten articles discussing the concept of health from the care workers perspective. The subtheme physical was represented in five of these articles.^
